# Hydrogen Bonds under Electric Fields with Quantum Accuracy

**DOI:** 10.1021/acs.jpca.5c01095

**Published:** 2025-04-29

**Authors:** Alessandro Amadeo, Marco Francesco Torre, Klaudia Mráziková, Franz Saija, Sebastiano Trusso, Jing Xie, Matteo Tommasini, Giuseppe Cassone

**Affiliations:** † Department of Chemistry, Biology and Biotechnologies, University of Perugia, Via dell’Elce di sotto, 8, 06123 Perugia, Italy; ‡ Department of Chemical, Biological, Pharmaceutical and Environmental Science, University of Messina, 98166 Messina, Italy; § J. Heyrovský Institute of Physical Chemistry, Czech Academy of Sciences, Dolejškova 3, 18223 Prague, Czechia; ∥ Institute for Chemical-Physical Processes, National Research Council of Italy (CNR-IPCF), 98158 Messina, Italy; ⊥ Ministry of Education Key Laboratory of Cluster Science, Beijing Key Laboratory of Photoelectronic/Electrophotonic Conversion Materials, School of Chemistry and Chemical Engineering, Beijing Institute of Technology, Beijing 100081, P. R. China; # Dipartimento di Chimica, Materiali e Ing. Chimica “G. Natta”, Politecnico di Milano, Piazza Leonardo da Vinci, 32, 20133 Milano, Italy

## Abstract

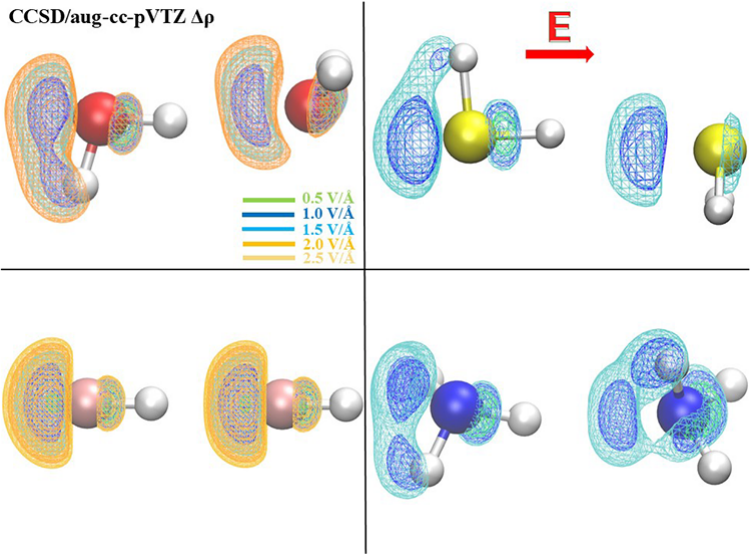

Hydrogen bonds (H-bonds) are pivotal in various chemical and biological systems and exhibit complex behavior under external perturbations. This study investigates the structural, vibrational, and energetic properties of prototypical H–bonded dimers, water (H_2_O)_2_, hydrogen fluoride (HF)_2_, hydrogen sulfide (H_2_S)_2_, and ammonia (NH_3_)_2_ – and the respective monomers under static and homogeneous electric fields (EFs) using the accurate explicitly correlated singles and doubles coupled cluster method (CCSD) for equilibrium geometries and harmonic vibrational frequencies and the perturbative triples CCSD(T) method for energies. As for the vibrational response of the H_2_O, HF, H_2_S, and NH_3_ monomers, it turns out that dipole derivatives primarily govern the geometry relaxation. Perturbation theory including cubic anharmonicity can reproduce CCSD results on the vibrational Stark effect, except for NH_3_, where deviations arise due to its floppiness. The field-induced modifications in H-bond lengths, vibrational Stark effects, binding energies, and charge-transfer mechanisms in monomers and dimers are elucidated. Symmetry-adapted perturbation theory (SAPT) analysis on dimers reveals that electrostatics dominates the stabilization of H-bonds across all field strengths, while induction contributions increase significantly with stronger fields, particularly in systems with more polarizable atoms. Our results reveal a universal strengthening of intermolecular interactions at moderate to strong field intensities with significant variability among dimers due to inherent differences in molecular polarizability and charge distribution. Notably, a direct correlation is observed between the binding energies and the vibrational Stark effect of the stretching mode of the H-bond donor molecule, both in relation to the charge-transfer energy term, across all of the investigated dimers. All of these findings provide insights into the EF-driven modulation of H-bonds, highlighting implications for catalysis, hydrogen-based technologies, and biological processes.

## 1 Introduction

Although the hydrogen bond (H-bond) was discovered more than a century ago, such an intermolecular bond remains among the most fascinating and elusive topics in chemical physics.^1^ Beyond the evidence that H-bonded condensed phase systems like water typically exhibit a rich tapestry of anomalous and intricate properties,^2^ the interest around the H-bond is deeper and essentially rooted in its fundamental and ubiquitous nature in hydrogen-bearing polar systems.

According to the IUPAC definition,^3^ a typical H-bond is represented as X–H···Y–Z. Under this configuration, an attractive effective interaction, in which there is evidence of bond formation, occurs between a hydrogen atom from a donor molecule or a molecular fragment X–H in which X is more electronegative than H, and an atom Y or a group of atoms Y–Z in the same or a different molecule. Presently, the nature of H-bonds remains a subject of debate,^4^ with ongoing discussions generally involving qualitative traditional concepts such as “covalency” and “electrostatic” degrees.^5^ Although these concepts are employed in an attempt to identify strong (i.e., short) and weak (i.e., long) H-bonds in liquids and solids, the H-bond comprises electrostatic attraction, polarization, dispersion, and partial covalency,^6^ the latter commonly expressed in terms of charge transfer. Besides the evidence that only recently developed experimental techniques have been capable of measuring charge transfer and associated quantum effects in water H-bonds,^7^ these ingredients altogether give rise to the embryo of the intermolecular bonding at the basis of life,^8^ and of a myriad of applications.^9−11^ Furthermore, H-bonds play a pivotal role in shaping the structures of biomolecules and orchestrating biochemical processes. From the intricate folding of proteins^12^ to the replication of DNA, H-bonds serve as molecular glue, holding biomolecular structures together and tuning their functions,^13^ while the manipulation of H-bonds offers promising eco-friendly solutions in technological apparatus.^14^

In recent years, the advent of techniques such as scanning tunneling microscopy (STM) and atomic force microscopy (AFM) has enabled the manipulation of H-bonds at the molecular scale.^15−17^ By applying tailored electric fields (EFs), new scenarios have been disclosed in catalysis, demonstrating the potential of these fields to drive various chemical reactions.^18−21^ Moreover, computational studies have elucidated the intricate interplay between external EFs and H-bonds in H-bonded systems.^22−26^ Using advanced computational methods, some of us have unraveled the subtle nuances of how EFs modulate the dynamics of H-bond networks in bulk liquid water,^27^ in ammonia,^28^ and aqueous ammonia mixtures,^29,30^ shedding light on phenomena ranging from phase transitions and electrophoresis to molecular dissociation and proton transfers.^27−33^

The capability of EFs in finely modulating H-bonds is rooted in the complex interactions they establish with electrons and protons. This way, chemical and H-bonded systems are significantly susceptible to local EFs found in condensed phases.^34−36^ The fluctuations of molecular dipoles in liquid water are known to produce fields larger than 1 V/Å,^37^ whereas in aqueous solutions^38−40^ and in the presence of solvated ions,^41,42^ local field intensities exceeding 2–3 V/Å are ubiquitous. Comparable EF strengths, spontaneously generated by charge separation, endow water microdroplets with surprising catalytic power,^43−46^ even though recent investigations have mitigated such a claim.^47,48^ Last but not least, superficial EFs present on the catalytic surfaces of TiO_2_ have been recently indicated as responsible for peculiar arrangements of H-bonded water molecules that are capable of boosting green hydrogen production.^49^

The spectroscopic response of simple molecular systems,^50−52^ and extended bulk H-bonded,^23,26,28^ water systems has been thoroughly investigated by means of density functional theory (DFT) approaches in the context of vibrational Stark spectroscopy, in which frequency shifts induced by intense fields to selected vibrational modes are conveniently exploited as local probes^34^. An impressive recent study^53^ has explored the accuracy of several DFT methods for simple (a)polar molecules placed under external intense EFs. Although density functionals usually employed for identifying molecular geometries or electronic energies are known to suffer from the delocalization error^54^ – also when molecules are exposed to EFs^55−57^ – Scheele and Neudecker,^53^ show that “DFT methods can be used for accurate calculations in oriented external electric fields”, at least at the single-molecule level. On the other hand, when H-bonds are present and charge transfer, dispersion, and polarization effects are considered, the agreement between DFT computations and higher-level calculations afforded with explicitly correlated methods seems to get worse in the water case.^58^ Therefore, to better understand how prototypical H-bonded dimers react to external EFs, here, we report a computational study on the dimers of water, hydrogen fluoride, hydrogen sulfide, and ammonia (along with the respective monomers) under static and homogeneous EFs.

## 2 Methods

In the current work, the molecular properties of the water, hydrogen fluoride, hydrogen sulfide, and ammonia molecules, as well as of their dimers, subjected to external electric fields (EFs) were investigated with the explicitly correlated coupled cluster methods using the quantum chemistry software Gaussian 16^59^ and PSI4 v1.9.1.^60^ We adopted the singles and doubles coupled cluster method (CCSD)^61−64^ for the optimized molecular geometries and harmonic vibrational frequencies, and we used the perturbative triples method CCSD(T),^65−67^ for the energies. Also, we evaluated the CCSD geometries of H-bonded dimers in the absence of an electric field against their CCSD(T) counterparts (see the Supporting Information, SI).

CCSD geometries and CCSD(T) energies were computed for both the dimers and monomers. All main calculations were carried out by employing the augmented correlation consistent Dunning’s aug-cc-pVTZ basis set. Furthermore, extrapolation to the complete basis set (CBS) limit was performed based on energies from CCSD(T) calculations employing three different basis sets: aug-cc-pVDZ, aug-cc-pVTZ, and aug-cc-pVQZ using two different extrapolation schemes (i.e., Helgaker et al.^68^ and Halkier et al.^69^). Such a procedure was executed for both the monomers and the dimers and for all of the investigated field strengths. To enforce a fixed reference system, we applied the external static and homogeneous EF toward the direction identified by the X–H (X = F, O, S, N) covalent bond donating the intermolecular H-bond of each dimer, which we define as the *x* Cartesian axis. The reference system was kept unaltered upon application of the field by means of the *NoSymm* keyword in the Gaussian 16 input. For the monomers, we applied the EF along the direction of the dipole. In this case, the reference was taken in such a way that positive values of the field correspond to the positive direction of the dipole (which is defined from the negative to positive charge).

We used symmetry-adapted perturbation theory (SAPT)^70^ to assess contributions of interaction energy components (electrostatics, exchange-repulsion, induction, and London dispersion) in H-bonded dimers by using the PSI4 code. The calculations were conducted at the recommended SAPT2+(3)δMP2/aug-cc-pVTZ level of theory, which is considered the “gold standard” of SAPT.^71^ The SAPT2+(3)-ct/aug-cc-pVTZ level was used to evaluate the charge-transfer component, which is extracted from the induction term.^72^ Full SAPT profiles computed at the SAPT2+(3)-ct/aug-cc-pVTZ level of theory are shown in Figure S6 of the SI. The external EF was applied along the positive direction of the X–H covalent bond donating the H-bond, as described above, by employing the *perturb_dipole [x, y, z]*, *perturb_h True* and *perturb_with dipole* keywords. Geometries of H-bonded dimers optimized at the CCSD/aug-cc-pVTZ level were used for the SAPT analyses. The SAPT2+(3)δMP2/aug-cc-pVTZ total interaction energies were compared to the reference CCSD(T)/CBS interaction energies for all the investigated EF intensities to assess the accuracy of the chosen SAPT level with applied EF (see SI for details).

All of the dimers investigated here present different stationary points on their own potential energy surfaces. As an example, the water dimer shows 10 Smith stationary points,^73^ whereas the hydrogen sulfide dimer exhibits at least 12 distinct stationary points.^74^ For the (H_2_O)_2_ and (H_2_S)_2_ systems, we investigated their respective global potential energy surface minima. In the water dimer, this structure corresponds to the well-known nonplanar configuration,^75^ which was found to be the most stable one from pioneering CCSDTQ calculations.^75^ In such a dimer structure, the plane spanned by the hydrogen bond donor molecule is perpendicular to the plane spanned by the H-bond acceptor H_2_O species. Moreover, the OH covalent bond of one water molecule is almost aligned with the H-bond it is donating.

The global minimum of the hydrogen sulfide dimer partially resembles that of water, even though it is energetically less separated from the other stationary points on the potential energy surface.^74^ Its *C*_s_ structure exhibits a relatively weak H-bond and, similar to the case of water described above, the planes spanned by the H_2_S molecules are orthogonal to each other. For distinct reasons, the scenario characterizing the structural arrangements of the (HF)_2_ and (NH_3_)_2_ moieties is drastically different. Energetics associated with the conformers of the hydrogen fluoride dimer clearly indicate that the nonlinear arrangement is more stable than the linear and the cyclic ones, although the difference is smaller than 2 kcal/mol.^76^ Such evidence would suggest employing the nonlinear configuration as the starting point of our calculations. However, in light of the relatively small energy barrier – which further reduces when considering the zero-point energy,^76^ – and since the primary effect of the application of strong external EFs is that of aligning the molecular dipole moments, we decided to use the linear (HF)_2_ conformer, where both dipoles and the H-bond are almost aligned along the same direction of the external field (see below).

The situation of the ammonia dimer is more subtle. It is well-known that its potential energy surface is quite flat, which leads to a substantial floppiness of all the dimer structures associated with the stationary points.^77^ This implies that all conformers become essentially either metastable or unstable under the action of external perturbations. Hence, for consistency with the other molecules, we decided to start our calculations from the stationary point corresponding to the linear staggered structure (see Figure 1 in ref (77)), so as to have a shared reference system across all the investigated H-bonded dimers.

**Figure 1:**
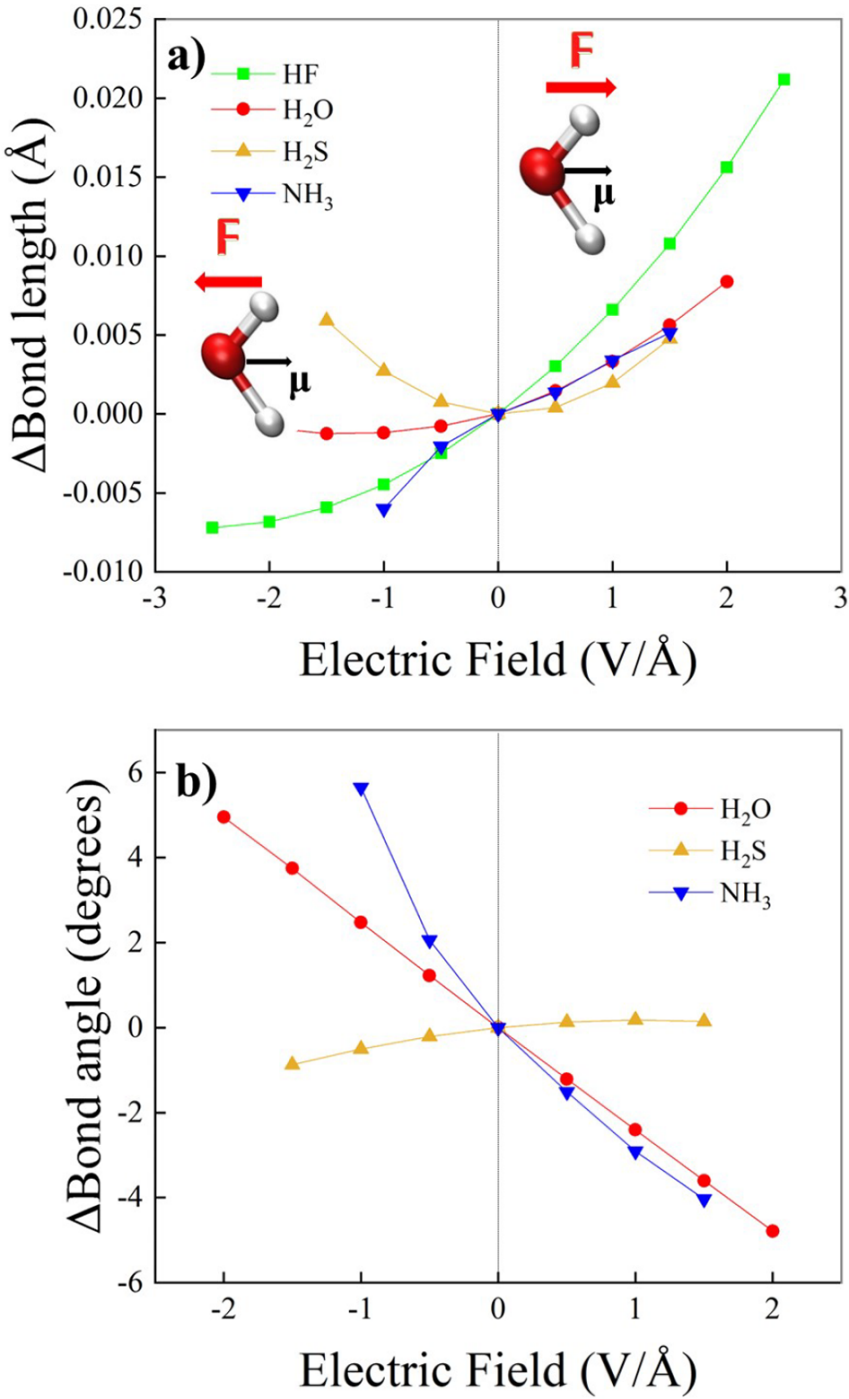
Electric-field-induced variation relative to the zero-field case of the covalent bond length (a) and molecular angle (b) of hydrogen fluoride (green squares), water (red dots), hydrogen sulfide (yellow up-triangles), and ammonia (blue down-triangles) for various field intensities and evaluated at the CCSD/aug-cc-pVTZ theory level. Positive (negative) values of the field strength correspond to cases in which the field axis is aligned toward (against) the molecular dipole vector (see the inset in (a) for water). Vertical dotted lines separate the two distinct field-molecule arrangements here investigated.

The top panels of Figure 9 report a sketch of the overall molecular geometries adopted for the calculations on the dimers with the applied external field. The calculations on the H_2_O, H_2_S, HF, and NH_3_ monomers were carried out by aligning the respective dipole moment vectors along with the *x*-axis, the direction toward which the external field was oriented, as displayed in the inset of Figure 1 for the case of water. Similarly to the dimer case, the use of the *NoSymm* keyword allowed for fixing the overall reference system.

Even though for the dimers the EF was applied along the positive direction of the X–H covalent bond donating the H-bond (i.e., from the donor to the acceptor molecule, corresponding in our reference to positive values of the field), for the monomers we also investigated fields oriented against the molecular dipole moment (which in our reference corresponds to negative values of the applied field). Such a choice was dictated by the fact that, whereas these polar dimers are generally very susceptible to fields aligned against the H-bond (which correspond to fields partially aligned against both dipole moment vectors), the monomers appeared to be quite resilient even under strong fields applied in the opposite direction of their dipole moment (in the range ± ∼1.5 to 2.5 V/Å). This way, EFs ranging from 0.0 up to ∼1.5 to 2.5 V/Å were applied depending on the convergence of the electronic wave function. For the dimers, field intensities beyond ∼1.5 to 2.5 V/Å induced serious instabilities that made it impossible to achieve the convergence of the wave function. Following previous results obtained by our group,^58^ we selected an EF step increment of 0.5 V/Å.

## 3 Results and Discussion

### 3.1 Monomers

As detailed in the Methods section, we performed all monomer calculations with the dipole moment vectors aligned along the *x*-axis, which coincides with the positive direction of the external field, as shown in Figure 1a for water. Several aspects of the field-molecule interactions can be emphasized by considering the effect of an EF on the bond length and molecular angle for the four investigated monomers. The results are presented in Figure 1 as the difference between the EF-applied and zero-field values. As observed in Figure 1a, the bond length exhibits a monotonic increase for all monomers when the EF is applied along the respective dipole moment vector. Hydrogen fluoride (HF) exhibits a pronounced sensitivity to the application of the external EFs (green squares). Hydrogen sulfide (H_2_S) shows a symmetrical response regardless of the EF’s direction (yellow up-triangles), while water (H_2_O) follows a roughly similar trend as H_2_S for positive EFs only but appears to be less affected by negative ones (red dots). The ammonia (NH_3_) monomer, while adhering to the trends of H_2_O and H_2_S for positive EFs, is notably more influenced by negative EFs, where a monotonic decrease is still observed, but it is featured by an evident point of inflection at 0 V/Å (blue down-triangles). Figure 1b displays the impact of the field on the bond angle of the monomers, with the trivial exception of HF. While the bond angle of H_2_O changes linearly as a function of the field strength, NH_3_ exhibits a seemingly 2-fold trend depending on the mutual orientation of the EF and the molecular dipole vector. In fact, fields oriented against the latter influence to a larger extent the molecular geometry with respect to the effect that the EF has when applied parallel to the dipole, a circumstance in line with the result shown in Figure 1a. Notably, the H_2_S bond angle is the least affected by the application of static and homogeneous EFs, exhibiting only very small variations with respect to the zero-field value.

As discussed in ref (78), under the influence of external fields, the molecular structure relaxation can be approximated, at first order, by collective nuclear displacements *q*_*k*_ along the *k* = 1 ··· 3*N* – 6 normal modes of the molecule:
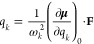
1where ω_*k*_ is the angular frequency of the given *k*th mode and (*∂***μ**/*∂q*_*k*_)_0_ is the dipole derivative along the *k*th mode, evaluated in the absence of the applied field ***F*** (as usual, the origin of the normal coordinates corresponds to the zero-field equilibrium position, for which *q*_*k*_ = 0). The data reported in Figure 1 can be qualitatively interpreted based on eq 1 since the quantities appearing in eq 1 can be determined from the Gaussian output of a typical frequency calculation. We report in Table 1 a summary of the numerical data for the symmetric stretching and bending modes of the four examined molecules. Such modes have been selected because the relaxation along their normal coordinates can be qualitatively related to the change in X–H bond length (X = F, O, S, N) and H–X–H valence angle (X = O, S, N). Indeed, the slopes of the bond length changes induced by the applied field follow the increasing trend H_2_S < NH_3_ ≈ H_2_O < HF, which follows qualitatively the increasing trend of the dipole derivatives with respect to the X–H stretching modes reported in Table 1. Similarly, the trend exhibited by the slope of the field-induced change of the valence angle (Figure 1b), namely H_2_S < H_2_O < NH_3_, follows qualitatively the increasing trend of the dipole derivatives with respect to the bending modes reported in Table 1. This correspondence between the slopes of the trends in Figure 1 and the dipole derivatives reported in Table 1 can be interpreted based on eq 1, which establishes a linear relation between the geometry relaxation described by a given normal mode *q*_*k*_ (e.g., stretching or bending) and the applied field ***F***. Based on Table 1, we observe that the vibrational frequencies of the stretching mode vary across the molecules comparatively less than the dipole derivatives (the same is true also for the bending modes). Therefore, the dependence of the *q*_*k*_ relaxation vs the applied field is mostly explained by the dipole derivatives, which is why the trend of the slopes of Figure 1 correlates with the trend of the dipole derivatives in Table 1, after properly considering the signs reported in Table 2.^79^

**Table 1:** Absolute Values of the Dipole Derivatives Computed at Zero Field for the Totally Symmetric Stretching Modes (*a*) and Bending Modes (*b*) of the Molecules Investigated in This Work^a^

molecule	*ν̅* (cm^–1^)	|(*∂*μ_*x*_/*∂q*_*k*_)_0_| (debye Å^–1^ amu^–1/2^)
H_2_S	2733^(a)^	0.07
NH_3_	3497^(a)^	0.31
H_2_O	3853^(a)^	0.33
HF	4169^(a)^	1.61
H_2_S	1221^(b)^	0.14
H_2_O	1659^(b)^	1.32
NH_3_	1067^(b)^	1.79

a Results are from the CCSD/aug-cc-pVTZ frequency calculations. The conversion factor from debye Å^–1^ amu^–1/2^ to atomic units (as in Table 2) is 4.876 × 10^–3^. The *x*-axis is oriented along the molecular dipole. Since all the reported modes belong to the totally-symmetric irreducible representation, the dipole derivatives are oriented along the symmetry axis of the molecules, which corresponds to the *x*-axis, i.e., (∂μ_*y*_/*∂q*_*k*_)_0_ = (∂μ_*z*_/*∂q*_*k*_)_0_ = 0.

**Table 2:** First and Second Derivatives of the Dipole and Polarizability Computed at Zero-Field for the Totally Symmetric Stretching and Bending Modes of the Molecules Investigated in this Work^a^

molecule	*ν̅*_*k*_ (cm^–1^)	ω_*k*_ (au)	*f*_*kkk*_ (au)	*∂μ*_*x*_/*∂q*_*k*_ (au)	*∂*^2^μ_*x*_/*∂q*_*k*_^2^ (au)	*∂*α_*xx*_/*∂q*_*k*_ (au)	*∂*^2^α_*xx*_/*∂q*_*k*_^2^ (au)
H_2_O	3853	1.755 × 10^–2^	–1.910 × 10^–5^	–1.605 × 10^–3^	6.151 × 10^–5^	0.117	1.150 × 10^–3^
NH_3_	3497	1.593 × 10^–2^	–1.189 × 10^–5^	1.505 × 10^–3^	3.793 × 10^–5^	0.104	0.137 × 10^–3^
H_2_S	2733	1.245 × 10^–2^	–0.721 × 10^–5^	0.341 × 10^–3^	3.138 × 10^–5^	0.178	1.598 × 10^–3^
HF	4170	1.900 × 10^–2^	–3.286 × 10^–5^	–7.868 × 10^–3^	0.774 × 10^–5^	0.136	3.624 × 10^–3^
H_2_O	1659	7.561 × 10^–3^	–7.778 × 10^–7^	6.423 × 10^–3^	6.859 × 10^–5^	–2.150 × 10^–^^2^	1.658 × 10^–3^
NH_3_	1067	4.862 × 10^–3^	–9.755 × 10^–7^	8.739 × 10^–3^	15.62 × 10^–5^	2.120 × 10^–2^	1.599 × 10^–3^
H_2_S	1221	5.565 × 10^–3^	–0.808 × 10^–7^	–0.683 × 10^–3^	–1.820 × 10^–5^	–3.496 × 10^–2^	1.760 × 10^–3^

a Results are from CCSD/aug-cc-pVTZ calculations and are reported in atomic units^80^ to ease the numerical evaluation of eq 2. The *x*-axis is oriented along the molecular dipole. The stretching modes are the first four lines; the bending modes are the last three lines. Since all the reported modes belong to the totally symmetric irreducible representation, the dipole derivatives are oriented along the symmetry axis of the molecules, which corresponds to the *x*-axis, i.e., (∂^2^μ_*y*_/*∂q*_*k*_^2^)_0_ = (*∂*^2^μ_*z*_/*∂q*_*k*_^2^)_0_ = 0. Further details about the calculation of these quantities are reported in the Supplementary Information.

**Figure 2:**
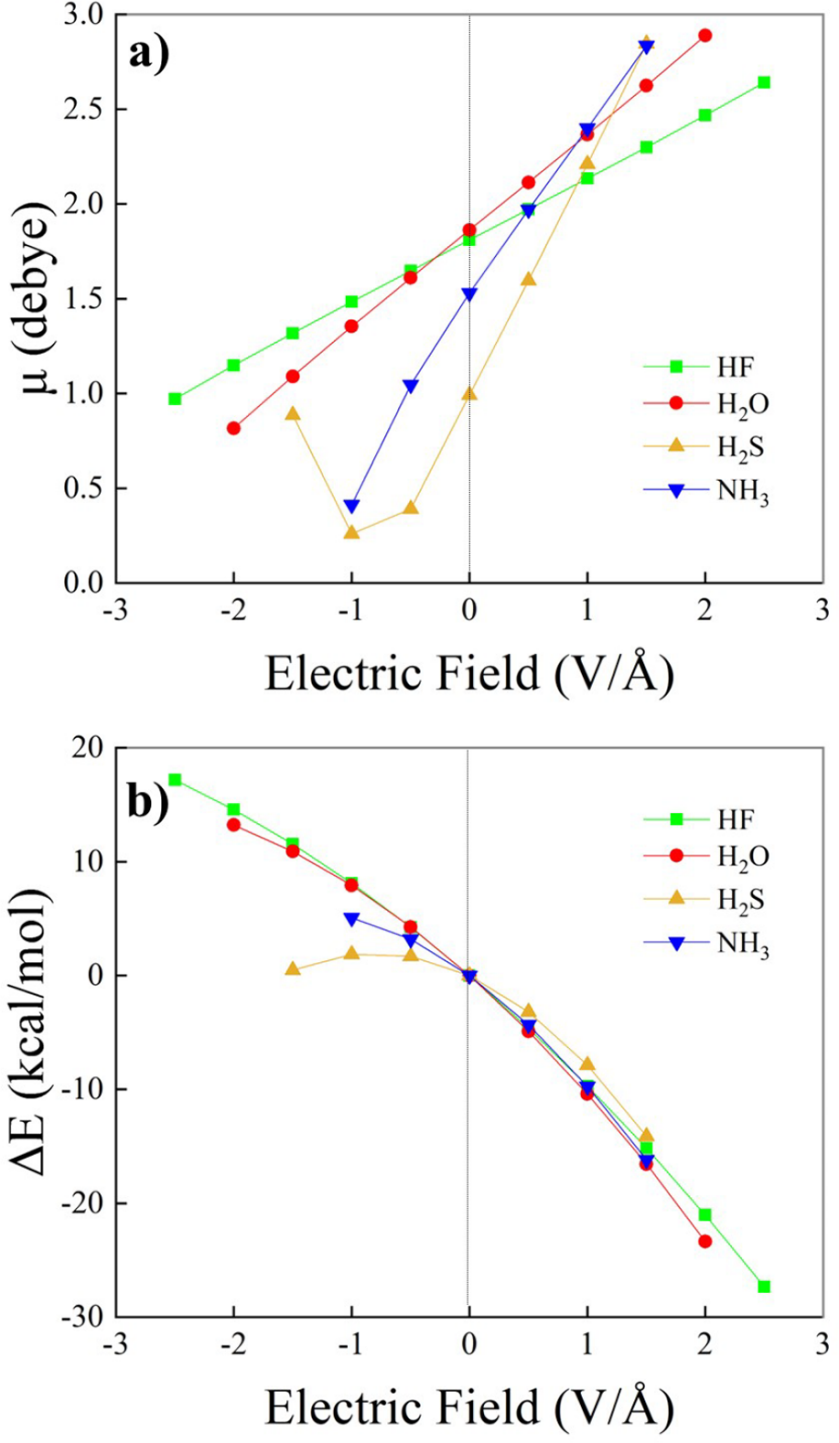
Dipole moment (a) and ground-state energy relative to the zero-field case (b) of the investigated monomers as a function of the electric field intensity evaluated at the CCSD/aug-cc-pVTZ theory level.

Figure 2a shows the effect of EF on the dipole moment of each monomer. Interestingly, HF and H_2_O exhibit a linear response to the application of the EFs within the range of strength here explored, although at more extreme field strengths, fully nonlinear effects were reported by some of us for the water monomer.^58^ On the other hand, NH_3_ and especially H_2_S show clearly nonlinear responses of the respective dipole moments to negative EFs. Due to the coupling of the external field and the molecular dipoles, the total Hamiltonian of the systems is modified from *Ĥ*_0_ (i.e., at zero-field) to *Ĥ* = *Ĥ*_0_ – **μ̂** ·***F***, where **μ̂** is the dipole moment and ***F*** is the EF vector. By virtue of the Hellmann–Feynman theorem, it follows that the expectation value of the dipole moment in the presence of the field is related to the change of the ground-state energy Δ*E* = ⟨*Ĥ*_0_⟩–⟨**μ̂**⟩·***F*** of the system, i.e., dΔ*E*/d*F* = – ⟨**μ̂**⟩·***u***_*F*_, where ***u***_*F*_ is the unit vector aligned along the EF. Therefore, when the EFs that are applied opposite to the molecular dipole are strong enough to displace the molecular charge distribution up to the point that the resulting expectation value of the electric dipole ⟨**μ̂**⟩ vanishes, the potential energy surface Δ*E*(***F***) reaches a stationary point. As shown in Figure 2b, this is located approximately around *E* = −1 V/Å for H_2_S, which displays for such a field strength a minimum value of the dipole approaching zero. For the other molecules, these stationary points are clearly located at more extreme field regimes, as witnessed by the incipient curvature of the respective ground-state energy curves in Figure 2b. This observation can be rationalized in the following way. By approximating the expectation value of dipole ⟨**μ̂**⟩ by a linear response through the molecular polarizability **α**_0_, we have ⟨**μ̂**⟩ = ⟨**μ̂**_0_⟩ + **α**_0_***F***. The critical value of the EF for which dipole ⟨**μ̂**⟩ vanishes is given by ***F***_*c*_ = – **α**_0_^–1^⟨**μ̂**_0_⟩. As discussed above, unless the polarizability tensor is significantly anisotropic, ***F***_*c*_ tends to be opposite to the natural dipole moment of the molecule ⟨**μ̂**_0_⟩. Clearly, molecules with small dipoles and large polarizability, such as H_2_S, tend to have smaller values of the critical field. For instance, the slopes at zero field in Figure 2a can be used to infer the relative importance of the polarizability of the different molecules, which leads to the following scale of decreasing polarizability: H_2_S > NH_3_ > H_2_O > HF.

**Figure 3:**
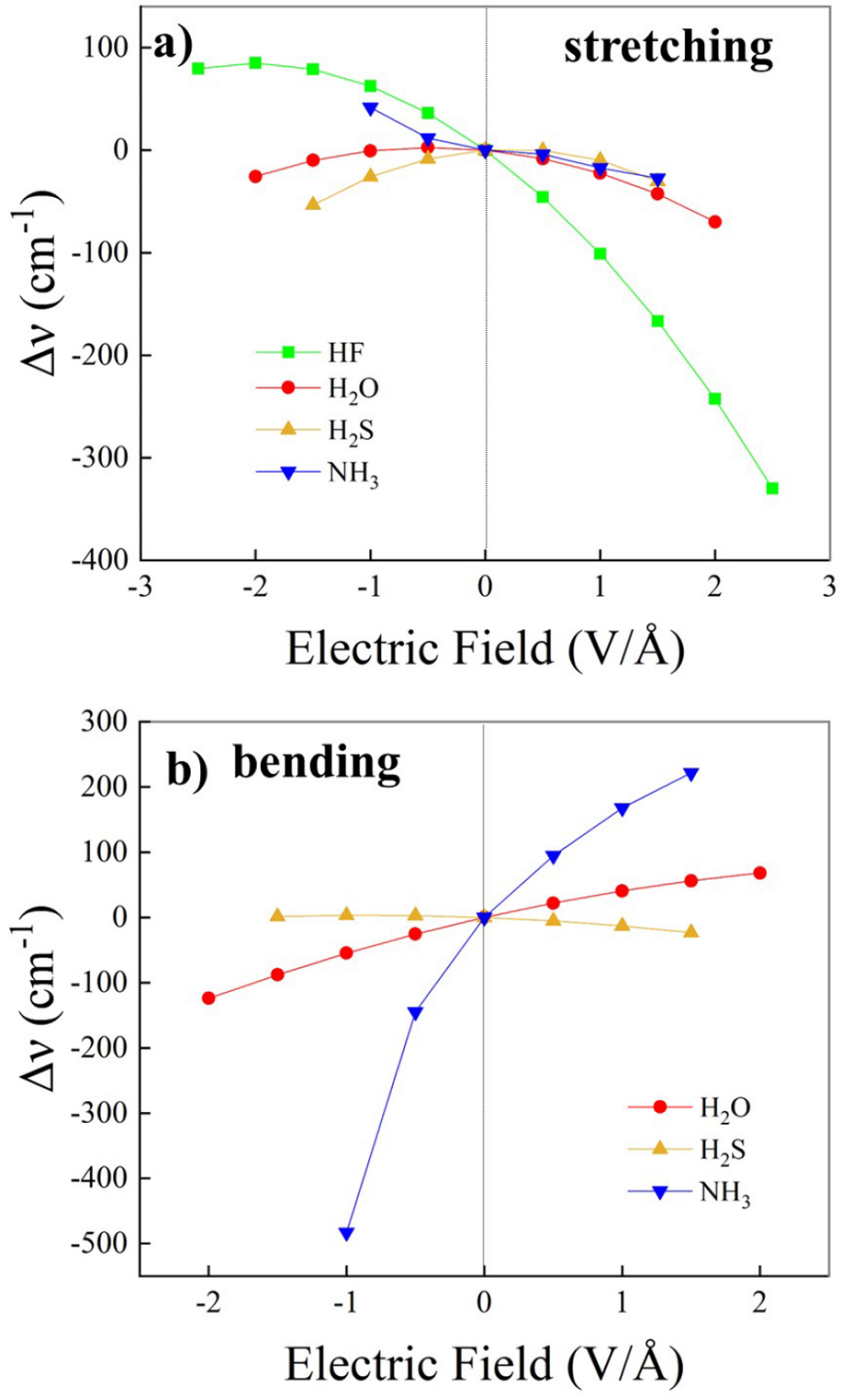
Infrared vibrational Stark effect of the symmetric stretching (a) and bending (b) vibrational modes of the investigated monomers evaluated at the CCSD/aug-cc-pVTZ theory level.

Upon application of the external electrical perturbation, the vibrational frequencies change. This effect is known as the vibrational Stark effect, and it is displayed in Figure 3 for all the investigated monomers. As evident in Figure 3, we observe a sizable dependence of the stretching and bending frequencies on the magnitude and direction of the applied EF. Notably, the red shift of the X–H stretching frequency (X = F, O, S, N) under a positive EF is significantly more pronounced for the HF monomer compared to that observed for the other cases. In contrast, an EF oriented against the dipole vector induces a red shift for H_2_O and H_2_S, while we observe an evident blue shift of the stretching mode of the HF and NH_3_ monomers. The frequencies of the bending mode (Figure 3b) under positive EFs are blue-shifted for H_2_O and NH_3_, whereas H_2_S exhibits a slight red shift. The significant Stark effect associated with the bending mode of NH_3_ is of particular relevance because of the floppiness of this molecule and its tendency toward exploration of out-of-plane angles, such as the well-known “umbrella inversion”. It is worth anticipating here that in more complex systems, the red- (blue-)shift of the stretching (bending) mode frequency is symptomatic of a potential strengthening of possible intermolecular bonds (e.g., H-bonds), as it will be laid out for the dimers.

In a pioneering work, Buckingham^81^ reports the first attempt to apply perturbation theory to investigate EF-induced frequency shifts, whereas 35 years later, Bishop applied perturbation theory to provide a more comprehensive theoretical analysis of the vibrational Stark effect,^82^ which can be used to rationalize the trends observed in our calculations. For a field applied along the direction of the molecular dipole (*x*) and for a normal mode *q*_*k*_ that produces a variation of the dipole just along the *x*-axis, the expression derived by Bishop can be written as follows:
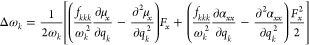
2where *f*_*kkk*_ is the zero-field cubic anharmonic term associated with the normal coordinate *q*_*k*_; the first and second derivatives of the dipole and polarizability are evaluated at zero-field at the equilibrium position (*q*_*k*_ = 0) – for notational simplicity, we omit the subscripts 0 in the derivatives. To assess the Stark effect described by eq 2, and compare it with the results from calculations under EFs (Figure 3), we computed the energy *E*, dipole μ_*x*_, and polarizability α_*xx*_ as a function of *q*_*k*_. By polynomial fitting of *E*(*q*_*k*_), μ_*x*_(*q*_*k*_), and α_*xx*_(*q*_*k*_), we obtained the terms required to numerically evaluate the right-hand side of eq 2. These are collected in Table 2 for the stretching and bending modes of the four molecules considered in this work. As detailed in the SI (see Figure S11a), Bishop’s equation accounts for the overall trends of Figure 3a, which is remarkable since eq 2 is derived from perturbation theory, and the applied EFs are quite strong. The deviations observed between the predictions of eq 2 and the data of Figure 3a can be ascribed to the increasing influence of higher-order terms, such as the quartic anharmonic term in the potential or the third-order dipole and polarizability derivatives. Interestingly, the cases of HF, H_2_O, and H_2_S are described by Bishop’s equation better than NH_3_, which is indeed the most challenging molecule due to the floppiness of the potential already mentioned. Among the four molecules, the steep trend in the Stark effect of HF stands out. The clue can be found in the values of *f*_*kkk*_ and *∂*μ_*x*_/*∂q*_*k*_, the largest in the series of molecules investigated here. Furthermore, *∂*^2^μ_*x*_/*∂q*_*k*_^2^ is the smallest in the series, which explains how for HF the coefficient of the linear term vs. the field, , is dominant and leads to the strongest Stark effect observed here. As for the vibrational Stark effect of the bending modes, Bishop’s equation can describe the finite field results of Figure 3b much better (see SI, Figure S11b). A possible explanation for the better performance can be found by looking at Table 2. For the bending modes, the anharmonicity terms *f*_*kkk*_ of the bending modes are 2 orders of magnitude smaller than those of the stretching modes. This can be taken as an indication that the higher-order terms that are neglected in Bishop's derivation are effectively more negligible for the bending modes than they are for the XH stretching modes. Under this condition, the slope of the Stark effect near zero field values can be approximated by
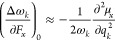
3Therefore, the values of the second-order derivatives of the electric dipole (Table 2) can be used to justify the decreasing trend of the slopes (NH_3_ > H_2_O > H_2_S) observed in Figure 3b. Indeed, *∂*^2^μ_*x*_/*∂q*_*k*_^2^ is largest for NH_3_, decreases by slightly more than a factor of 2 in H_2_O, and becomes slightly negative in H_2_S, which fully accounts for the trends of Figure 3b.

**Figure 4:**
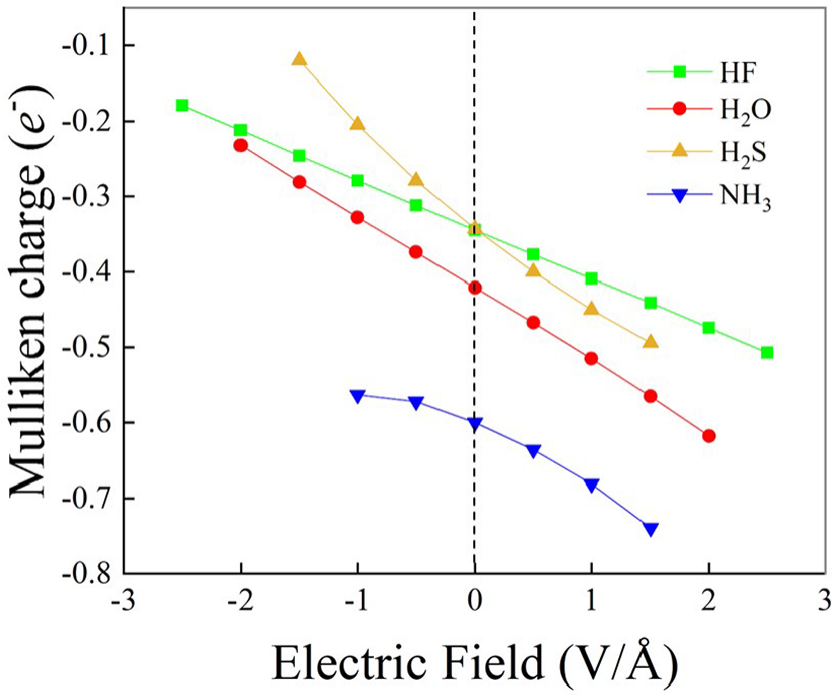
Mulliken charge localized on the heteroatoms (F, O, S, N) of hydrogen fluoride (green squares), water (red dots), hydrogen sulfide (yellow triangles), and ammonia (blue triangles) for various field intensities and evaluated at the CCSD/aug-cc-pVTZ theory level.

In the investigated monomers, the application of the EF also has a measurable impact on the Mulliken charges of the heteroatoms (F, O, S, and N), as reported in Figure 4. Although HF and H_2_O show a linear correlation between the EF intensity and the Mulliken charges localized on the respective heteroatoms, H_2_S and NH_3_ exhibit a nonlinear trend, a circumstance resembling the scenario encountered with the dipole moment (Figure 2a). Besides, the accumulation of larger fractions of (negative) Mulliken charges upon increasing the EF strength in the direction of the dipole moments indicates the propensity for the onset of charge-transfer phenomena at about 1–2 V/Å. Such an observation anticipates the possibility of field-induced H-bond strengthening and partial charge-transfer phenomena in the case of interacting molecules. It is noteworthy that for strong EFs applied against the H_2_S dipole moment direction (i.e., for −1.0 and –1.5 V/Å), the trend of the Mulliken charge on the sulfur atom does not match that recorded for the dipole moment and reported in Figure 2a. We surmise that this disagreement might be due to the localization failure of the electron density when field-induced dipole moment flipping is observed.

**Figure 5:**
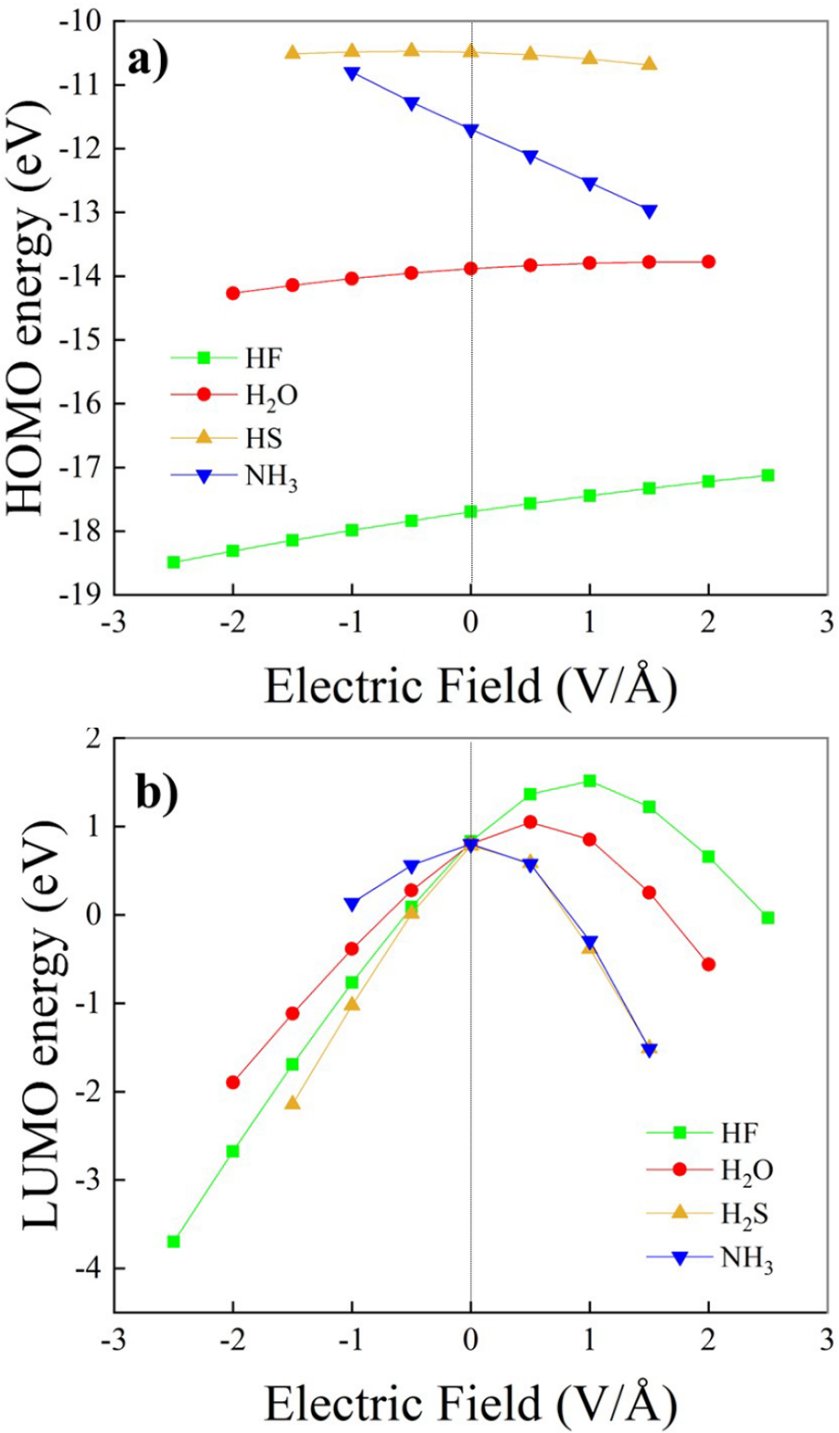
HOMO (a) and LUMO (b) energies of hydrogen fluoride (green squares), water (red dots), hydrogen sulfide (yellow up-triangles), and ammonia (blue down-triangles) for various field intensities and evaluated at the CCSD/aug-cc-pVTZ theory level.

Figure 5 illustrates the impact of the EF on the energies of the frontier molecular orbitals. Specifically, Figure 5a displays how the highest occupied molecular orbital (HOMO) energy depends on the externally applied EF strength and direction. While positive EFs lead to a slight but measurable increase in the HOMO energy for HF and H_2_O (positive slope), the NH_3_ monomer response is characterized by a sudden linear decrease in the HOMO energy as a function of the field (negative slope). Interestingly, the HOMO energy of the H_2_S monomer appears to be almost unaffected by the application of the EF, independent of its direction. Albeit the HOMO energy response for all the monomers is monotonic and almost linear (Figure 5a), a strongly nonmonotonic behavior as a function of the field is observed for the energy of the lowest unoccupied molecular orbital (LUMO, Figure 5b). While the LUMO energy of NH_3_ and H_2_S exhibits a superimposable decreasing trend under the action of positive EFs, the LUMO energy of HF and H_2_O increases at low-to-moderate field strengths and then decreases at more extreme regimes. Such a 2-fold behavior of the LUMO energy of HF and H_2_O, coupled with the similar monotonic increase of the HOMO energy (Figure 5a), indicates that though for moderate EF regimes the HOMO–LUMO energy gap is preserved, under stronger fields this gap narrows, as witnessed by the HOMO–LUMO energy gap as a function of the field displayed in Figure S7 of the SI. A similar rationale holds also for H_2_S and NH_3_, though in the latter case an opposite trend is recorded for the HOMO energy (Figure 5a). Finally, all monomers experience a monotonic decrease in the LUMO energy when subjected to progressively stronger EFs applied against the dipole moment vector, as shown in the left part of Figure 5b.

### 3.2 Dimers

**Figure 6:**
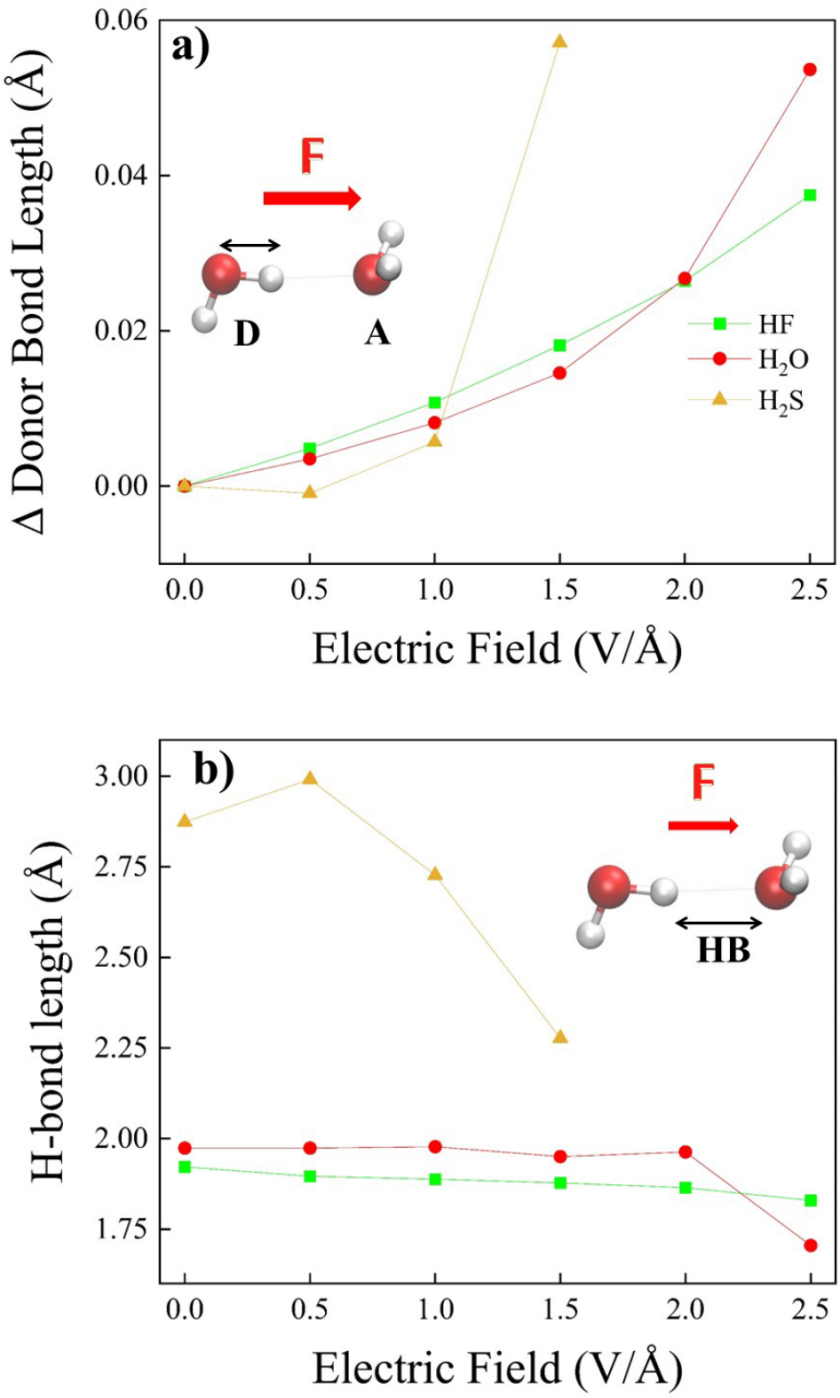
(a) Optimized X–H (X = F, O, S) covalent bond length of the H-bond donor molecule (D) lying on the H-bond of the investigated dimers and (b) the respective relaxed H-bond length as a function of the field strength evaluated at the CCSD/aug-cc-pVTZ theory level. In the insets, the considered molecular arrangement for the (H_2_O)_2_ moiety only and the respective plotted quantities are highlighted.

In bulk liquids, the application of external static and homogeneous electric fields (EFs) exceeding ∼0.1 V/Å induces fast reorientations of the molecular dipoles^22,23,25,26,83,84^. Such evidence testifies that strong interactions arise at the molecular level between the field and the electric moments of various orders, especially with the total dipole moment vector resulting from the superposition of the monomeric dipoles. Since the application of fields oriented against the dipole vectors promptly induces a global reorientation of all the investigated dimers, only the “positive” relative arrangement of the molecules with respect to the field has been considered in our analysis (this field orientation is shown in Figure 6 for (H_2_O)_2_). Besides, in the case of the ammonia dimer, we have observed a substantial instability of the intermolecular interactions even when the field was relatively weak and oriented along the positive direction of the overall dipole moment (see the SI for the results on the ammonia dimer). This complex behavior has to be ascribed to the peculiar potential energy surface (PES) of (NH_3_)_2_. In fact, it is well-known that the different conformers associated with the local minima of the PES of the ammonia dimer are separated by very low barriers, which makes such a system highly fluxional.^77^ This circumstance seriously hinders the analysis of stable molecular configurations of ammonia dimers under the influence of the EF. For this reason, we consider in the main text just the HF, H_2_O, and H_2_S dimers.

Among the 10 Smith stationary points^73^ of (H_2_O)_2_, the nonplanar geometry corresponding to the well-known global minimum configuration^85,86^ has been chosen. Under such a configuration, the oxygen–oxygen distance is equal to 2.93 Å at our CCSD/aug-cc-pVTZ level, a value in fairly good agreement with experimental results (i.e., 2.98 Å).^87,88^ The accordance with the experiment is even closer for the dipole moment; in fact, our estimate is equal to 2.64(85) D, a value almost exactly matching the experimental one of 2.64(29) D.^87^ Furthermore, the global PES minimum of (H_2_S)_2_ forming a weak but stable H-bond^89^ has been considered. Under such a molecular arrangement, the sulfur–sulfur distance estimated at the CCSD level is equal to 4.21 Å, a value slightly larger than the available experimental estimate (i.e., 4.11 Å).^89^ As far as the (HF)_2_ case is considered, a molecular geometry different from that associated with the global minimum has been analyzed. This choice has been dictated by the fact that the application of external EFs to the (HF)_2_ conformer associated with the well-known global minimum of the PES^90^ induces an alignment of the HF monomer dipoles along the field axis. This way, the latter geometry, which corresponds to a zero-field local minimum of the PES, has been selected for the calculations both in the absence and in the presence of the external EF. Such a choice leads to discrepancies between our calculated zero-field fluorine–fluorine distance and the experimentally derived one (i.e., 2.84 vs 2.72 Å,^91^ respectively). Finally, it is worth noticing that the evaluated CCSD geometries in the absence of the field are in fairly good agreement with the more accurate CCSD(T) ones, as reported in Table S1 of the SI. In particular, the CCSD(T) X–H (X = F, O, S, N) covalent bond lengths of the H-bond donor moiety are on average larger than the CCSD ones by 0.0033 Å. Oppositely, H-bonds are on average shortened by 0.037 Å in CCSD(T) geometry optimizations with respect to their CCSD counterparts.

As shown in Figure 6a, the application of a field aligned along the X–H (X = F, O, S) covalent bond donating the intermolecular H-bond in the HF, H_2_O, and H_2_S dimers leads to an overall elongation of the bond length. Nevertheless, at the minimum field intensity here explored (i.e., 0.5 V/Å), we observe for (H_2_S)_2_ an initial slight reduction of the S–H covalent bond length with respect to the zero-field value. Interestingly, although at low-to-moderate field regimes we obtain the fastest relative elongation for (HF)_2_, at the highest field intensity (i.e., 2.5 V/Å) we observe a sudden increase in the O–H covalent bond length of the H-bond donor molecule in (H_2_O)_2_, which overcomes the trend of (HF)_2_ (Figure 6a).

Although the progressive increase of the external field strength leads to measurable variations of the molecular geometries of all of the investigated dimers, only the H-bond length characterizing the structure of (H_2_S)_2_ is significantly perturbed by the field, as displayed in Figure 6b. In fact, in (H_2_O)_2_ and (HF)_2_, we observe a substantial plateau of the intermonomeric distance as a function of the field intensity. Such a nontrivial finding might indicate that the H-bond strength in the (H_2_O)_2_ and (HF)_2_ dimers could be insensitive to the applied field, even at strong field regimes of 2 V/Å. However, a more sensitive measure of the strength of the intermolecular interactions can be obtained by using the vibrational Stark effect.

**Figure 7:**
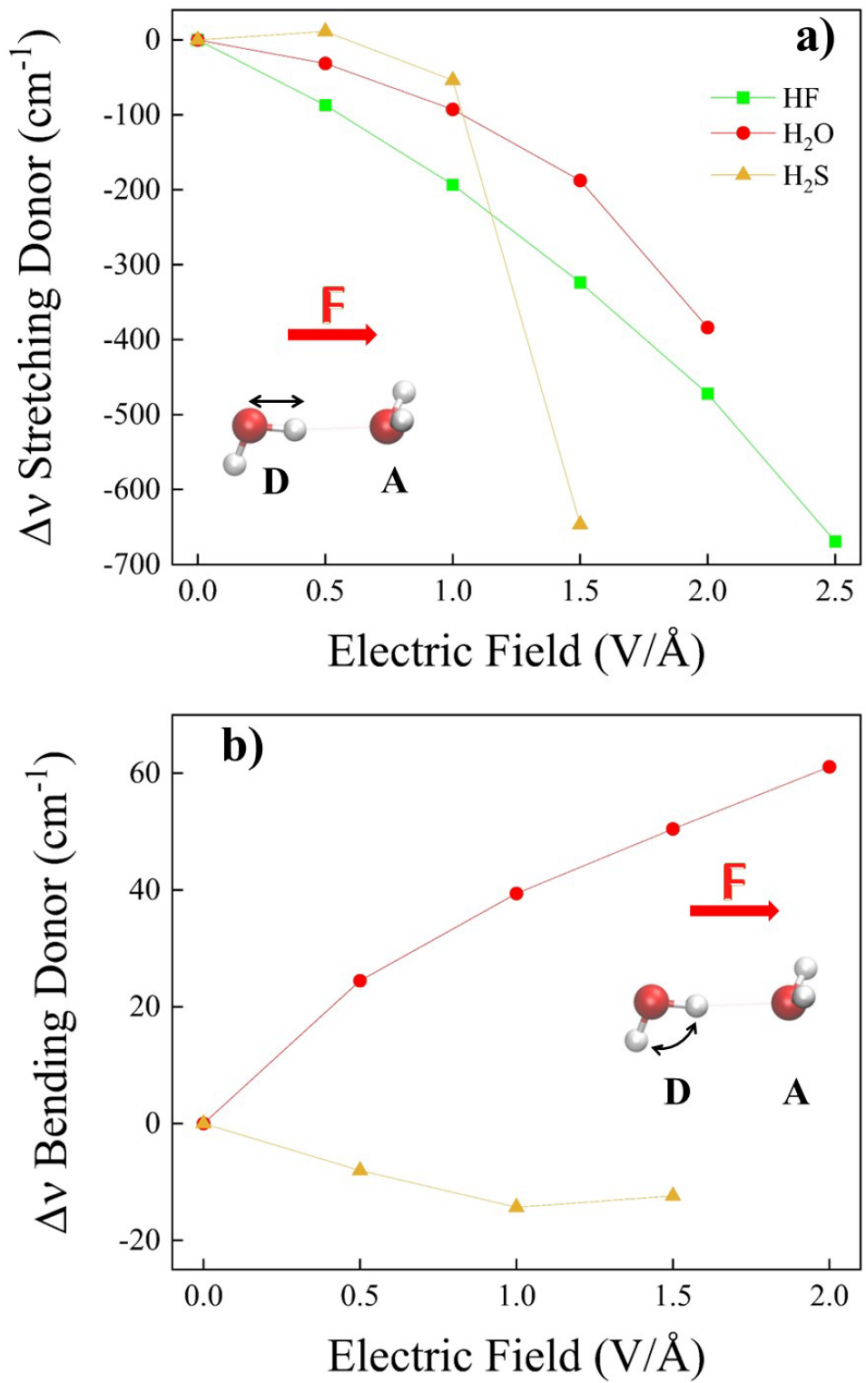
Infrared vibrational Stark effect of the symmetric stretching mode (a) of the X–H (X = F, O, S) covalent bond and bending (b) mode of the molecular species donating the H-bond in the investigated dimers (see legend) evaluated at the CCSD/aug-cc-pVTZ theory level.

To this aim, we have monitored the infrared (IR) vibrational frequencies associated with the symmetric stretching of the X–H (X = F, O, S) covalent bond donating the H-bond in the dimers (Figure 7a) and the bending frequency of the same H-bond donor molecule (Figure 7b), as depicted in the insets of Figure 7. Clearly, no bending mode is present for (HF)_2_. Similar to the previous findings, the response of (H_2_S)_2_ to the applied field deviates from that of (H_2_O)_2_ and (HF)_2_. In particular, while the vibrational Stark effect associated with the X–H stretching of (H_2_O)_2_ and (HF)_2_ shows a monotonic red-shift upon increasing the EF strength, we observe at 0.5 V/Å a slight blue-shift in the field-induced stretching frequency variation of (H_2_S)_2_ (Figure 7a). This suggests that whereas the net effect of the EF on the H-bond of (H_2_O)_2_ and (HF)_2_ is that of strengthening such an interaction, for (H_2_S)_2_ the application of the EF weakens the H-bond at the lower values of the field. Furthermore, the slope associated with the vibrational Stark effect in (HF)_2_ is the most negative one, a circumstance likely ascribed to the better coupling between the (fully aligned) system’s dipole moment vector and the external electrostatic field.

Contrary to the Stark effect of the X–H stretching, a blue shift of the bending indicates a field-induced strengthening of the intermolecular interactions.^23,27^ Indeed, this is observed for water (Figure 7b). However, for the HSH bending mode of the monomer donating the H-bond in (H_2_S)_2_, we find an opposite trend compared to that of the water dimer, and we observe in Figure 7b an overall red shift of the bending mode over the whole field strength range here explored. Moreover, as shown in Figure 7a, after the initial blue-shift of the S–H stretching mode at 0.5 V/Å, a global red-shift of the stretching frequency is observed at 1.0 V/Å and very prominently at 1.5 V/Å. This evidence shows in (H_2_S)_2_ a vibrational response to the external EF that is more complex than that in (H_2_O)_2_, which, however, indicates an overall strengthening of the intermolecular H-bond.

**Figure 8:**
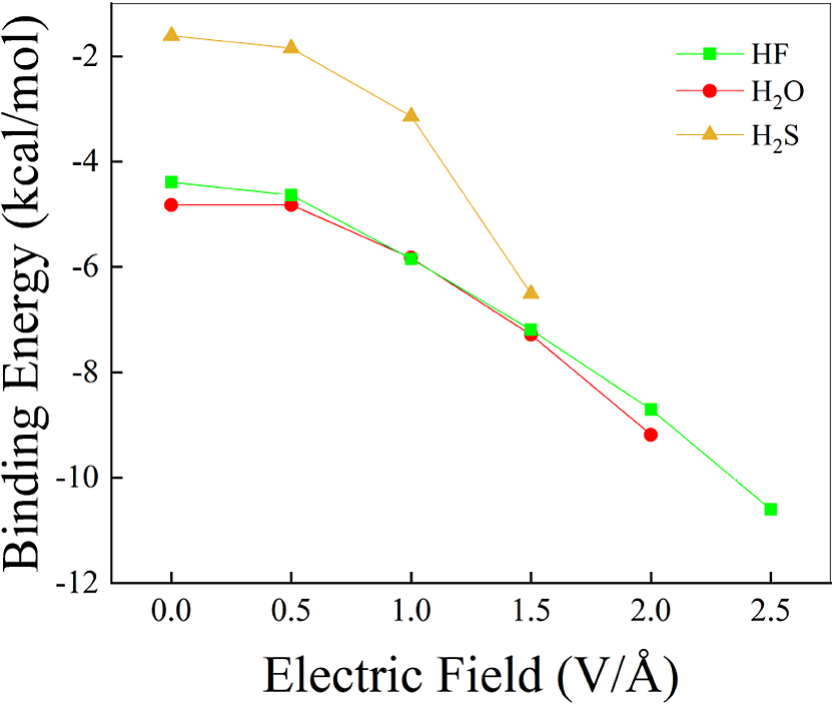
Binding energy associated with the H-bond of the hydrogen fluoride (green squares), water (red dots), and hydrogen sulfide (yellow up-triangles) dimers as a function of the electric field strength and evaluated up to the Complete Basis Set (CBS) limit determined by extrapolating the results stemming from CCSD(T) energy calculations employing the aug-cc-pV[X = 2, 3, 4]*Z* basis sets (i.e., CCSD(T)/CBS).

Notwithstanding the qualitative and quantitative differences observed for the vibrational Stark effect, the application of an external field strengthens the intermolecular interactions in all of the dimers and over the entire range of field strengths, as indicated by the computed binding energies shown in Figure 8. Somehow surprisingly, although the red-shift of the frequency of the X–H stretching mode is typically associated with the strengthening of the intermolecular interactions, this does not seem to be the case for fields on the order of 0.5 V/Å, where only a very modest (absolute) increase of the binding energy is observed, except for water. Indeed, a marginal weakening of the H-bond is triggered in (H_2_O)_2_ by an external field of 0.5 V/Å. Since in H-bonded bulk systems fields of this order of magnitude are certainly capable of making much more robust the three-dimensional network of H-bonds,^23,24,27^ we argue that this result is due to the lack of many-body dynamical phenomena in simple dimeric systems. On the other hand, EFs greater than 0.5 V/Å manifestly strengthen the H-bonds in all of the investigated dimers. Interestingly, the qualitative trend of the binding energies reported in Figure 8 correlates with the trend of the IR vibrational Stark effect of the symmetric stretching mode of the covalent bond donating the H-bond shown in Figure 7a. Such evidence suggests that charge transfer events, which potentially stabilize the dimers, might be in place at moderate-to-strong EF regimes, as discussed below.

**Figure 9:**
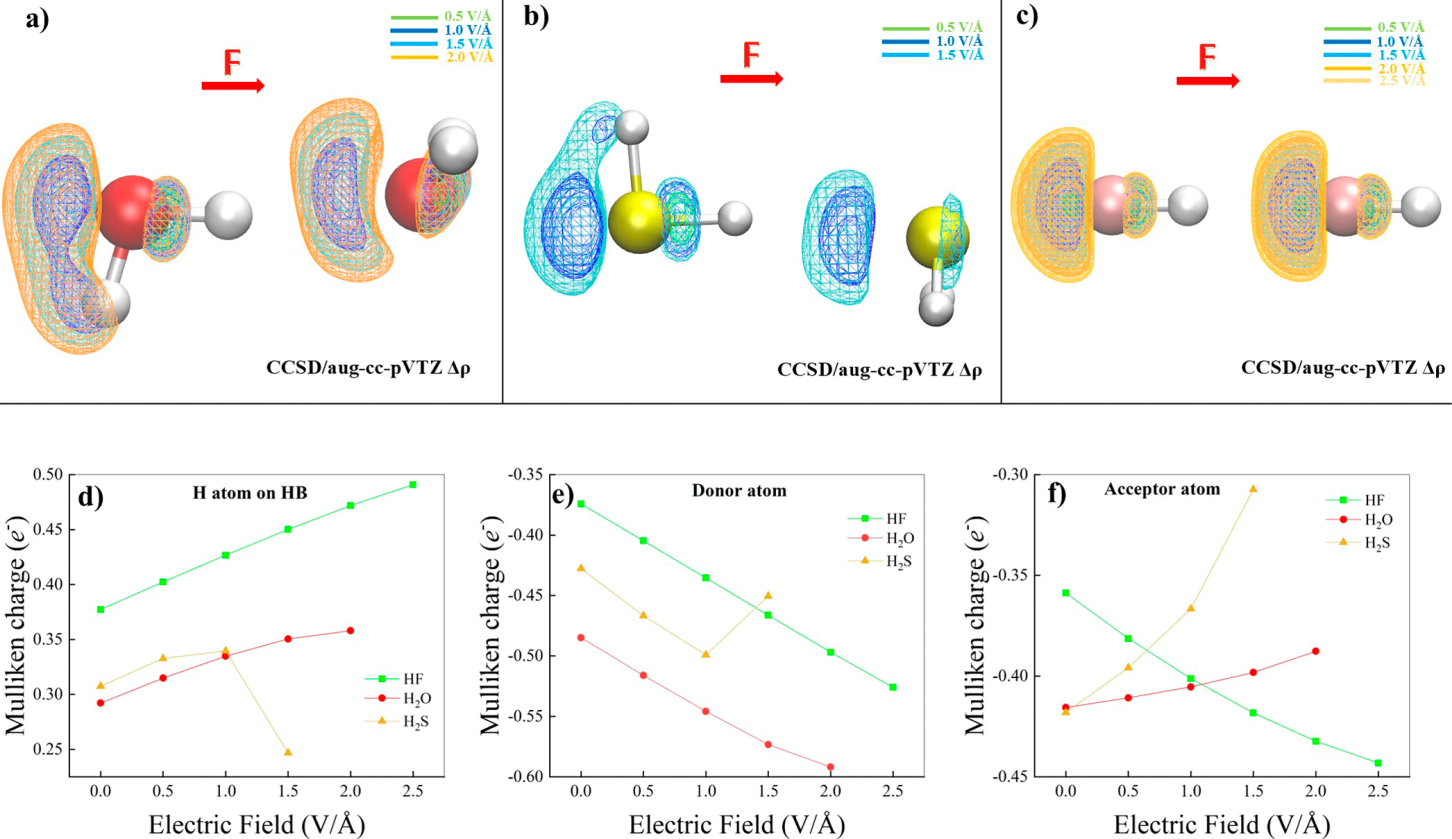
CCSD/aug-cc-pVTZ electron density differences (Δρ) for various field strengths (see legends) determined by using the zero-field density and nuclear positions as a reference for the water (a), hydrogen sulfide (b), and hydrogen fluoride (c) dimers. Isocountours correspond to Δρ = +0.003 au in all cases. In the bottom panels, the Mulliken charge localized on the hydrogen atoms lying on the H-bond (d), on the H-bond donor heteroatoms (e), and on the H-bond acceptor heteroatoms (f), are shown for the hydrogen fluoride (green squares), water (red dots), and hydrogen sulfide (yellow up-triangles) dimers as a function of the electric field strength.

As displayed in the top panels of Figure 9, progressively stronger EF strengths can displace in the H-bonded dimers more conspicuous electron density fractions (Δρ) over larger spatial domains. Besides the obvious field-induced shift of the electron density associated with the lone pairs of the H-bond donor molecules, relevant variations of ρ take place in the internuclear region spanned by the pair of heteroatoms of each dimer. In particular, a measurable fraction of the electron density migrates from the hydrogen atoms lying on the H-bond in all the dimers toward the covalently bound heteroatom (i.e., O, S, and F), a circumstance leading to a more polarized covalent bond donating the H-bond. The field-induced effect on the electron density in the H-bond region is spatially more prominent, and in the top panels of Figure 9a progressive Δρ increase is observed in the H-bond region for increasing EF strengths. The combined effect of the higher polarized state of the H-bond donor covalent bond, and the increment in electron density in the spatial region where the H-bond is located, gives rise to the overall field-induced strengthening of the H-bond itself discussed in Figure 8.

In an attempt to project the electron density on the nuclear positions, we have determined the Mulliken charges for all the investigated dimers at various EF intensities, as reported in the bottom panels of Figure 9. The charge of the hydrogen atom lying on the H-bond (Figure 9d) anticorrelates with the charge of the heteroatom of the H-bond donor molecule (Figure 9e), and this behavior is consistently observed in the three H-bonded dimers. However, a more intricate response is observed for the Mulliken charge of the H-bond acceptor heteroatom (Figure 9f). While the H-bond acceptor oxygen atom of (H_2_O)_2_ is almost insensitive to the field variation due to a compensating effect (i.e., the charge lost toward the H-bond is almost balanced by that coming from the two covalently bound hydrogen atoms), a fully discordant trend of charge vs. field is exhibited by the (HF)_2_ and (H_2_S)_2_ species. This observation can be justified as follows. The alignment of the dipole moment of the H-bond acceptor HF molecule with the external field (Figure 9c) allows for an increment in the local electron charge on the fluorine atom originating from the covalently bound hydrogen atom (Figure 9f). Conversely, the orthogonal orientation of the dipole vector of the H-bond acceptor H_2_S moiety with respect to the field axis (Figure 9b) hinders the efficient charge transfer toward the sulfur atom, resulting in a net decrement of the electron population (Figure 9f). Notably, at a field strength of 1.5 V/Å, the sudden decrease of the Mulliken charge on the hydrogen atom donating the H-bond (Figure 9d) and the sudden increase of the charge around the covalently bonded sulfur atom in the (H_2_S)_2_ species are symptomatic of a potential partial electron transfer event in this dimer.

**Figure 10:**
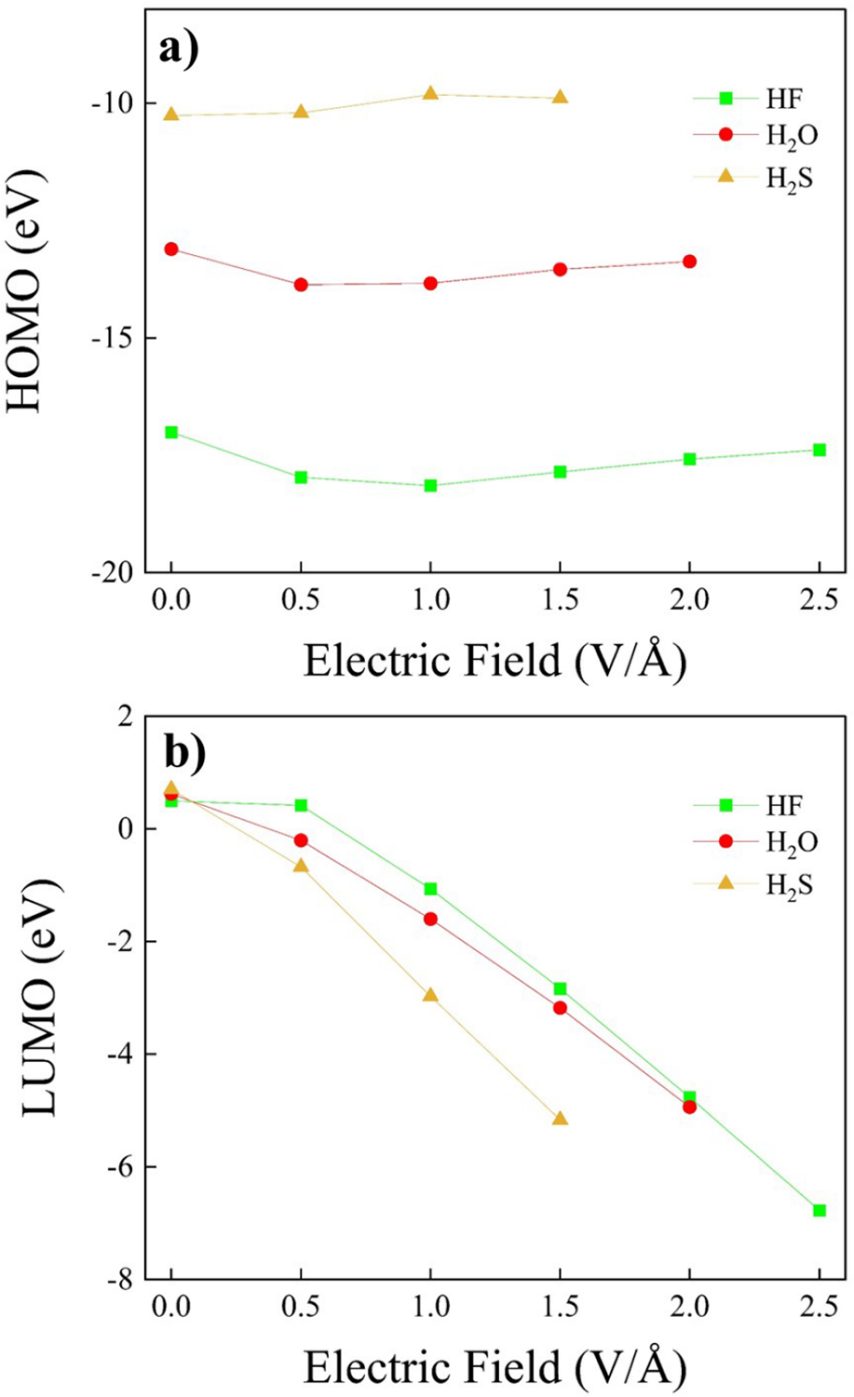
HOMO (a) and LUMO (b) energies of the hydrogen fluoride (green squares), water (red dots), and hydrogen sulfide (yellow up-triangles) dimers for various field intensities and evaluated at the CCSD/aug-cc-pVTZ theory level.

Although more difficult to interpret with respect to the monomer counterparts, we have determined the HOMO and LUMO energies for all of the dimers at various field intensities (Figure 10). Whereas the impact of the EF on the HOMO energies is somehow negligible (Figure 10a), a strong decrease of the LUMO energies is observed for increasing applied fields (Figure 10b): in all cases, a monotonic decrease from positive to negative energies is observed upon increasing the field strength, a circumstance that leads to a significant field-induced reduction of the HOMO–LUMO gap, as displayed in Figure S8 of the SI.

To understand the driving forces responsible for the discussed H-bond strengthening upon EF application, we have performed symmetry-adapted perturbation theory (SAPT)^70^ analysis. In SAPT, interaction energy is computed as a sum of physically meaningful interaction energy contributions, i.e., electrostatics (*E*_elect_), London dispersion (*E*_disp_), induction (*E*_ind_), and exchange-repulsion (*E*_exch–rep_):
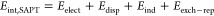
4

Note that the binding energy of dimers (*E*_BE_) includes interaction energy *E*_int_ and deformation energy *E*_def_, where the latter is associated with the geometry relaxation upon dimerization. Thus, the binding energy can be expressed as
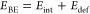
5where
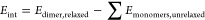
6

Thus, the interaction energy corresponds to the fraction of binding energy related to the interaction between isolated monomers adopting the geometries they exhibit in the dimers (i.e., unrelaxed geometries of monomers taken from the relaxed equilibrium structure of the H-bonded dimers). In the presence of an external EF, *E*_*BE*_ involves an additional contribution associated with the rearrangement of the molecular species under the field action.

**Figure 11:**
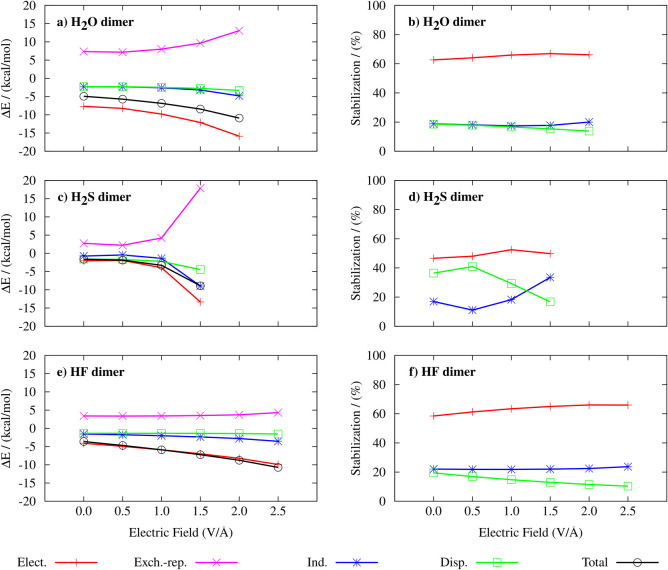
Dependence of the symmetry-adapted perturbation theory (SAPT) interaction energy components such as electrostatics (Elect.), exchange-repulsion (Exch.-Rep.), induction (Ind.), and London dispersion (Disp.) on the electric field intensity for water (a, b), hydrogen sulfide (c, d), and hydrogen fluoride (e, f) dimers. The left panels show absolute values of the SAPT interaction energy components while the right panels depict the contributions of the stabilizing components to the total stabilization energy of the dimers.

Overall, for all three dimers, the magnitude of all of the SAPT interaction energy components, together with the total interaction energy, increases with increasing EF, as reported in Figure 11. Among the stabilizing terms (electrostatics, induction, and London dispersion), electrostatics is the strongest one in all three dimers and for all the investigated field intensities. We have evaluated the contributions of the stabilizing components to the total stabilization in percentages (right side of Figure 11). Electrostatics dominates the stabilization in the absence as well as in the presence of the EF since its contribution is ∼50% or more in the whole range of field intensities and for all the dimers. Its importance slightly increases with an increase in the field intensity.

The induction term is rooted in polarization and charge-transfer phenomena. Since it is directly associated with the polarization of the electron cloud of one monomer caused by the action of the spontaneous EF generated by the other monomer and the subsequent interaction between them, the induction contribution to the overall stabilization is expected to increase upon increasing the external field. Although its contribution to stabilization is around ∼20% (i.e., less than electrostatics), its percentage increases more significantly upon increasing the EF than that of the other terms, which is best visible for the H_2_S dimer. In net contrast, the contribution of the London dispersion to stabilization decreases as a function of the field strength in all three cases. While at zero or low-to-moderate EF intensities, dispersion is similar to induction, or even more important than induction, as in the case of the H_2_S dimer, it becomes less significant at larger fields. The strengthening of the stabilizing interaction energy components with field intensity is partially compensated for by exchange-repulsion, i.e., the only destabilizing term here, arising when electron densities significantly overlap. Note that for H_2_S, electrostatics and induction decrease in absolute value at 0.5 V/Å and then start increasing at 1.0 V/Å and rise more steeply compared to electrostatics and induction of other dimers. This behavior is consistent with some of the previously discussed data, e.g., the increase of the H-bond length at 0.5 V/Å (Figure 6b) and the red-shift of the stretching frequency of the H-bond donor molecule (Figure 7a). The observation that H_2_S exhibits the most significant changes under the influence of the field could be related to the fact that the sulfur atom represents the largest acceptor among the analyzed dimers and, thus, is the most polarizable one. In contrast, the HF dimer shows the flattest response to the external EF: it exhibits only marginal changes in exchange-repulsion and London dispersion terms, and the increase in interaction energy can be attributed mainly to electrostatics. This, on the other hand, coincides with the fact that fluorine is the most electronegative atom and therefore keeps the electron cloud closer than oxygen or sulfur do. A comparison of stabilizing SAPT components for H_2_S and H_2_O at zero field shows that while electrostatics substantially dominates the H-bond interaction in H_2_O, it is only slightly more important than London dispersion for H_2_S. The weaker electrostatics and more significant London dispersion are related to the fact that the S atom is more polarizable than O, which consequently leads to the smaller directionality of the interactions in the H_2_S dimer compared to (H_2_O)_2_.^89^ This can probably also be transferred to other H-bonded systems containing S or O H-bond acceptors, as found for interaction between thiophosphate vs phosphate and uracil nucleobase.^92^

**Figure 12:**
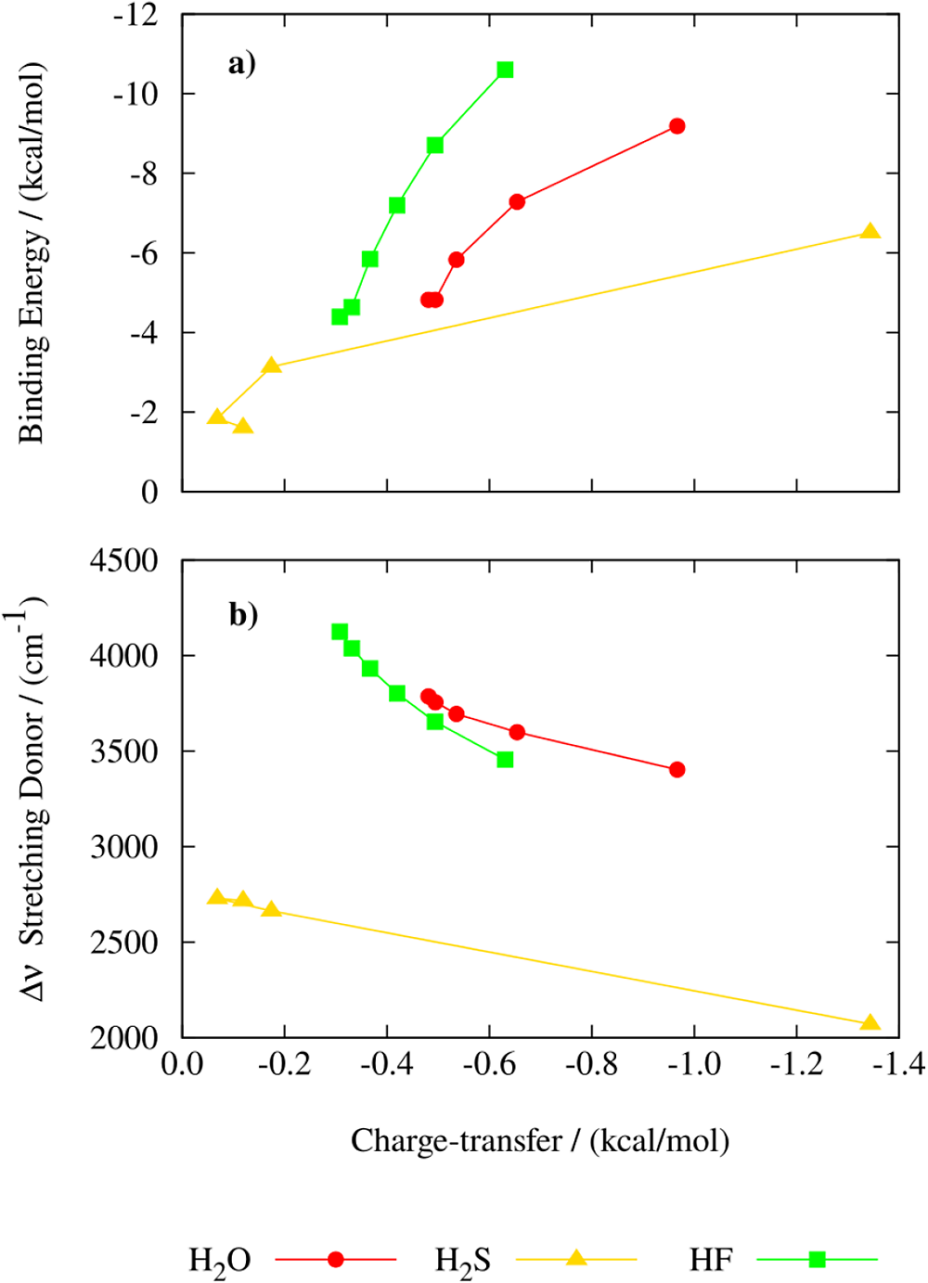
Relationship between binding energy (a) and symmetric stretching frequency of the H-bond donor (b) with respect to charge-transfer obtained with the SAPT2+(3)-ct/aug-cc-pVTZ for water (red), hydrogen sulfide (yellow), and hydrogen fluoride (green) dimers.

Furthermore, we have evaluated the extent of the existing correlations as a function of the applied field, among the SAPT charge-transfer term (*E*_ct_) and the binding energy, and between *E*_ct_ and the vibrational Stark effect associated with the symmetric stretching frequency of the H-bond donor. As reported in Figure 12a, when the EF is switched on, we observe quite a consistent rising trend between the binding energy and charge transfer *E*_ct_. The stabilization of the analyzed H-bonded dimers resulting from charge transfer is almost negligible for relatively low field strengths, yet it becomes important at stronger field intensities, which is particularly visible for the H_2_S dimer. For some reason that we were not capable of addressing, we note that there is a small inconsistency between the point corresponding to the smallest binding energy, in the absence of the field, and the rest of the points, especially in the (H_2_S)_2_ case. As for the vibrational Stark effect, a substantial red-shift associated with the lengthening of the X–H (X = O, S, F) covalent bond of the H-bond donor occurs with increasing EF intensity, which directly correlates with the increase in absolute value of Δ*E*_ct_. The slopes of the curves are similar for the H_2_O and the H_2_S dimer, whereas for the HF dimer, it appears that a smaller change in ΔE_*ct*_ is associated with a more significant red-shift of the stretching mode compared to the other two dimers.

## 4 Conclusions

In this work, by using the explicitly correlated singles and doubles coupled cluster method (CCSD) for equilibrium geometries and harmonic vibrational frequencies and the perturbative triples CCSD(T) method for energies, we investigated the effects induced by the application of static and homogeneous electric fields (EFs) on prototypical H-bonded dimers and their respective monomers.

We have analytically investigated the effects of the externally applied EFs on the vibrational response of the H_2_O, HF, H_2_S, and NH_3_ monomers and compared them against accurate CCSD calculations. We observed that the vibrational frequencies of the stretching and bending modes vary across the molecules comparatively less than those of the dipole derivatives, implying that the dependence of the geometry (i.e., normal modes) relaxation from the applied field is mostly explained by the dipole derivatives. The vibrational Stark effect is more subtle, and it is the result of the interplay of several molecular quantities, as it is clarified by Bishop’s perturbation theory approach to this physical problem,^82^ which includes zero-field cubic anharmonic terms, along with the first and second derivatives of the electric dipole and polarizability. By applying Bishop’s theory, we were able to rationalize the observed results on the vibrational Stark effect. A remarkable accordance with the accurate CCSD data is recorded for H_2_O, HF, and H_2_S, while we observe measurable deviations for the NH_3_ molecule due to its known floppiness.

Whereas in the monomers the application of an external field toward the direction of the molecular dipole vector leads to only slight variations of the X–H (X = F, O, S, N) covalent bond length, in the H-bonded dimers, an EF applied along the X–H covalent bond donating the H-bond triggers measurable elongations of the X–H covalent bonds. Besides, the field-induced change of the H-bond length appears to be only modest, except for (H_2_S)_2_. The hydrogen fluoride and water dimers exhibit H-bond lengths that are almost insensitive to the applied fields, up to strong fields of 2.0 V/Å. Nevertheless, important molecular geometrical rearrangements of all of the dimers are induced by the external field, especially in the moderate-to-strong field regime (i.e., *E* > 1 V/Å).

We also investigated the vibrational Stark effect and its field dependence. We observed that the application of the field along the X–H covalent bond donating the H-bond induces an overall red-shift of the symmetric stretching modes of the H-bond donor molecules, with the notable exception of (H_2_S)_2_ at the lowest field intensity considered here (0.5 V/Å). Where observed, the red-shift of the symmetric stretching mode indicates an overall field-induced strengthening of the H-bond, a result consistent with the observed increase of the CCSD(T)/CBS binding energies of all the dimers. Besides, the bending modes of the H-bond donor molecules exhibit varied shifts, with water exhibiting the well-known blue shift and hydrogen sulfide showing an unexpected red shift, a circumstance that underscores the complexity of the vibrational responses and their intricate relationship with the field-induced energetic stabilization of the dimers.

We examined the effect of the applied fields on the electron clouds of the H-bonded dimers by determining the field-induced variations of the electron density (Δρ). This analysis reveals that the overall field-induced strengthening of the H-bond is caused by the highly polarized state in which the X–H bond of the H-bond donor lies, and by the increment in electron density in the spatial region where the H-bond is located. Consequently, the binding energies universally increase with field intensity, driven by enhanced electrostatic and induction contributions. Indeed, symmetry-adapted perturbation theory (SAPT) analysis highlights the dominance of electrostatics in stabilizing the dimers, with induction contributions becoming increasingly significant at higher field strengths. In comparison, the role of London dispersion diminishes, as it is clearly visible in the highly polarizable hydrogen sulfide, which shows the largest zero-field contribution from London dispersion among the analyzed dimers. Remarkably, hydrogen sulfide also exhibits the most substantial induction effects. Charge transfer, while modest at lower fields, becomes significant at stronger field intensities, reinforcing the H-bond of the investigated dimers, which is especially visible for the hydrogen sulfide dimer. By employing the SAPT analysis, we could directly correlate these charge transfer events with the increased stabilization energy and the vibrational Stark effect of the stretching mode of the molecule donating the H-bond in the dimers. This direct correlation is especially evidenced by the more polarizable dimers.

Thanks to accurate quantum-mechanical methods, the current study unveils several molecular and electronic details highlighting the potential utility of static EFs in tuning H-bonding interactions, paving the way for applications in molecular engineering and advanced material design. These findings hold the potential for enhancing EF-mediated catalytic processes and understanding field-driven phenomena in biological and chemical systems.

## Supporting Information Available

Comparison of CCSD and CCSD(T) molecular geometries of the dimers at zero field; results of the calculations on the ammonia dimer; SAPT vs CCSD(T)/CBS interaction energies; HOMO–LUMO band gaps for monomers and dimers; and modeling the Stark effect with Bishop’s perturbation theory (PDF)
