# Supplementary material for: Hydrogen Bonds under Electric Fields with Quantum Accuracy
Source: J Phys Chem A. 2025 Apr 29;129(18):4077–92. doi: 10.1021/acs.jpca.5c01095 (PMC12067437; doi:10.1021/acs.jpca.5c01095)
Supplement: Supplementary file 1 — jp5c01095_si_001.pdf [file jp5c01095_si_001.pdf]

Supporting Information
Hydrogen Bonds under Electric Fields with Quantum Accuracy

Alessandro Amadeo,[†,‡,#] Marco Francesco Torre,[‡,#] Klaudia Mráziková,[¶,§,#] Franz Saija,[¶] Sebastiano Trusso,[¶] Jing Xie,[‖] Matteo Tommasini,[*,⊥] and Giuseppe Cassone[*,¶]

†*Department of Chemistry, Biology and Biotechnologies, University of Perugia, Via dell'Elce di sotto, 8, 06123, Perugia, Italy*

‡*Department of Chemical, Biological, Pharmaceutical and Environmental Science, University of Messina, 98166 Messina, Italy*

¶*Institute for Chemical-Physical Processes, National Research Council of Italy (IPCF-CNR), 98158 Messina, Italy*

§*J. Heyrovský Institute of Physical Chemistry, Czech Academy of Sciences, Dolejškova 3, 18223 Prague, Czechia*

‖ *Ministry of Education Key Laboratory of Cluster Science, Beijing Key Laboratory of Photoelectronic/Electrophotonic Conversion Materials, School of Chemistry and Chemical Engineering, Beijing Institute of Technology, Beijing 100081, P. R. China*

⊥*Dipartimento di Chimica, Materiali e Ing. Chimica "G. Natta", Politecnico di Milano, Piazza Leonardo da Vinci 32, 20133 Milano, Italy*

#*These Authors contributed equally*

E-mail: matteo.tommasini@polimi.it; cassone@ipcf.cnr.it

Comparison of CCSD and CCSD(T) geometries

We evaluated the CCSD/aug-cc-pVTZ geometries of H-bonded dimers in the absence of electric field against their CCSD(T)/aug-cc-pVTZ counterparts. Since Gaussian16 software[1] exhibited convergence issues for geometry optimizations with the CCSD(T) method, we used ORCA 6.0[2] for this purpose. The evaluated geometrical parameters are those thoroughly analyzed in the main text, *i.e.*, the donor X-H covalent bond length and length associated with the H$\cdots$Y H-bond. These parameters, determined from the CCSD geometries, are almost identical in ORCA 6.0 and Gaussian16 up to the third digit for the donor X-H covalent bond length and up to the second digit for the H$\cdots$Y H-bond distance, as reported in Table S1. The only exception is the H-bond length of the HF dimer, which shows a difference of ∼0.02 Å. The CCSD(T) X-H (X = F, O, S, N) covalent bond lengths of the H-bond donor moiety are on average larger than the CCSD ones by 0.0033 Å. Oppositely, H-bonds are on average shortened by 0.037 Å in CCSD(T) geometry optimizations with respect to their CCSD counterparts. Combined altogether, these effects indicate a quite slight H-bond strengthening at the CCSD(T) level, with the CCSD method very slightly overestimating the H-bond lengths, as shown in Table S1.

Table S1: Donor X-H bond lengths and hydrogen bond lengths (in Å) for H_2O, H_2S, HF, and NH_3 dimers using different computational methods.

	H_2O		H_2S		HF		NH_3	
	X-H	H-bond	X-H	H-bond	X-H	H-bond	X-H	H-bond
CCSD (Gaussian)	0.964	1.97	1.342	2.87	0.921	1.90	1.015	2.32
CCSD (ORCA)	0.964	1.97	1.342	2.87	0.921	1.92	1.015	2.32
CCSD(T) (ORCA)	0.968	1.95	1.345	2.80	0.924	1.91	1.018	2.28

Results on the ammonia dimer

It is well established that the ammonia dimer $(NH_3)_2$ exhibits a remarkably flat potential energy surface (PES),[3] leading to a significant structural flexibility (*e.g.*, floppiness) and a

very wide palette of accessible configurations. Due to such a inherent metastability, the ammonia dimer demonstrates a pronounced sensitivity even to relatively weak external electric fields applied along the N-H covalent bond donating the H-bond in the dimer. As displayed

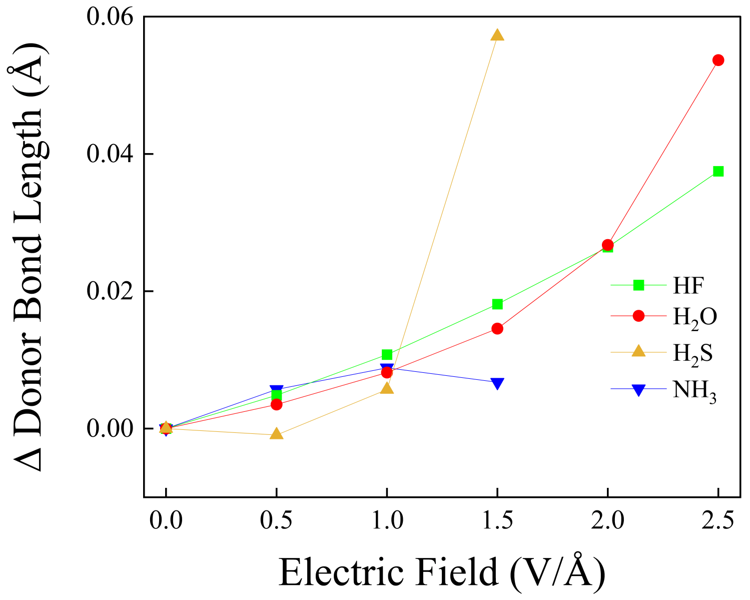

Figure S1: Optimized X-H (X=F,O,S,N) covalent bond length of the H-bond donor molecule lying on the H-bond of the investigated dimers (see legend) as a function of the field strength evaluated at the CCSD/aug-cc-pVTZ theory level.

in Fig. S1, when an electric field is applied parallel to the N-H bond involved in H-bonding, the covalent bond initially elongates, mirroring the behavior observed also in the HF, H_2O, and H_2S dimers discussed in the main text. However, at the highest field strength tested for $(NH_3)_2$ (*i.e.*, 1.5 V/Å), an unexpected shortening of the N-H bond occurs, a behavior drastically deviating from the trend observed in the other dimers (Fig. S1), suggesting that stronger electric fields have dramatic effects on the structural arrangement of the ammonia dimer. In net contrast, fields equal to or even beyond 1.5 V/Å do not produce any complete rearrangement of the molecular moieties involved in $(HF)_2$, $(H_2O)_2$, and $(H_2S)_2$. In fact, by inspecting the optimized stable structures, it is observed that all dimers preserves the H-bond whereas the monomers composing the $(NH_3)_2$ structure align their own dipole moments with the electric field axis, causing the cleavage of the internal H-bond in such a

dimer.

Such a scenario can be directly monitored by evaluating the distance between the H atom donating, at zero field, the H-bond and the acceptor atom X (X=F,O,S,N) as a function of the applied electric field, as reported in Fig. S2. Of course, such a distance corresponds to the H-bond length at all the explored field strengths for all dimers, with the exception of the ammonia one. As discussed in the main text, although a field-induced increase of

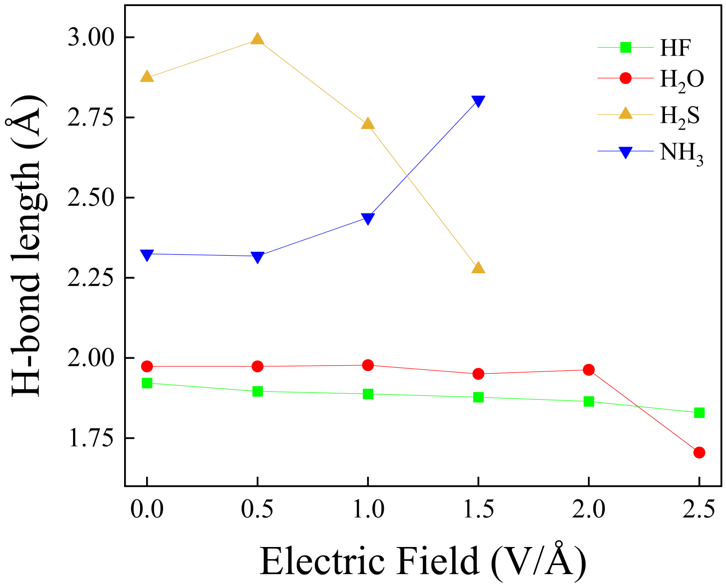

Figure S2: Relaxed distance between the H atom donating, at zero field, the H-bond and the acceptor atom X (X=F,O,S,N) of the investigated dimers (see legend) as a function of the applied electric field as a function of the field strength evaluated at the CCSD/aug-cc-pVTZ theory level.

the intermolecular distance in $(H_2S)_2$ is observed, an apparent initial insensitivity of the H-bond length is recorded at the lowest field intensity here explored (*i.e.*, 0.5 V/Å) for the reminder dimers. The dimers exhibiting the strongest H-bonds – such as $(HF)_2$ and $(H_2O)_2$ – are featured by a strong resilience of the H-bond length upon increasing the field intensity, being indeed characterized by an essentially flat response curve up to extreme fields. On the other hand, upon increasing the field strength beyond 1.0 V/Å, the distance identified by the closest H and X extramolecular atoms in the hydrogen sulfide and ammonia dimers shows opposite trends. In particular, such a distance between the NH_3 monomers in the

$(NH_3)_2$ structure, rather than shortening unexpectedly lengthens. This finding is rooted on the complete rearrangement of the molecular dipoles toward the field direction and the consequent breakage of the intermolecular H-bond, a feature not observed in the remainder dimers.

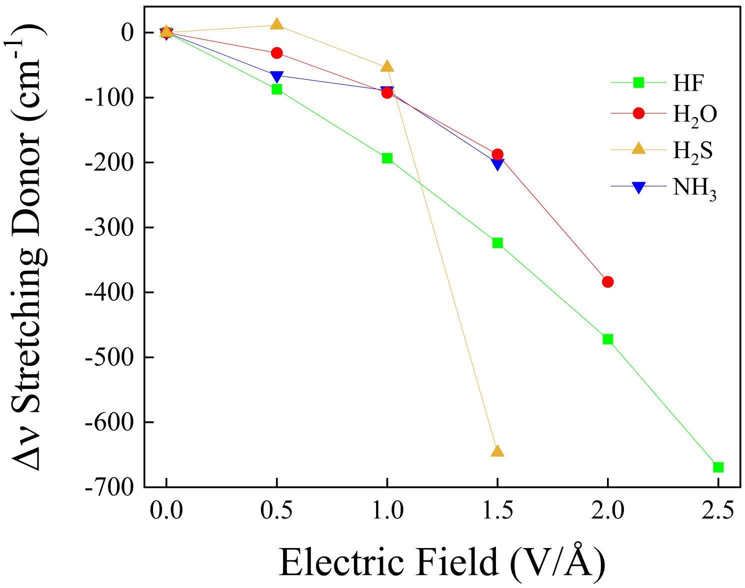

Figure S3: Infrared vibrational Stark effect of the symmetric stretching mode of the X-H (X=F,O,S,N) covalent bond of the molecular species donating the H-bond in the investigated dimers (see legend) evaluated at the CCSD/aug-cc-pVTZ theory level.

Additionally to structural changes, we investigated also the infrared (IR) vibrational frequencies associated with the symmetric stretching and bending modes of the X-H (X=F,O,S,N) covalent bond (*i.e.*, the H-bond donor), as reported in Fig. S3 and S4. The vibrational Stark effect associated with the N-H stretching mode exhibits a slight red-shift up to 1.0 V/Å, followed by a rapid increase at the highest field strength explore for $(NH_3)_2$ (*i.e.*, 1.5 V/Å), a trend fully consistent with the behavior observed in the HF, H_2O, and H_2S dimers (Fig. S3). Fig. S4 illustrates the Stark effect associated with the bending mode of the H-bond donor moiety for all the investigated dimers. As far as the response in the $(NH_3)_2$ case is concerned, an initial prompt blue-shift of the bending frequency is suddenly observed for a 0.5 V/Å field, followed by a much more marginal decrease and stabilization at 1.0 and 1.5

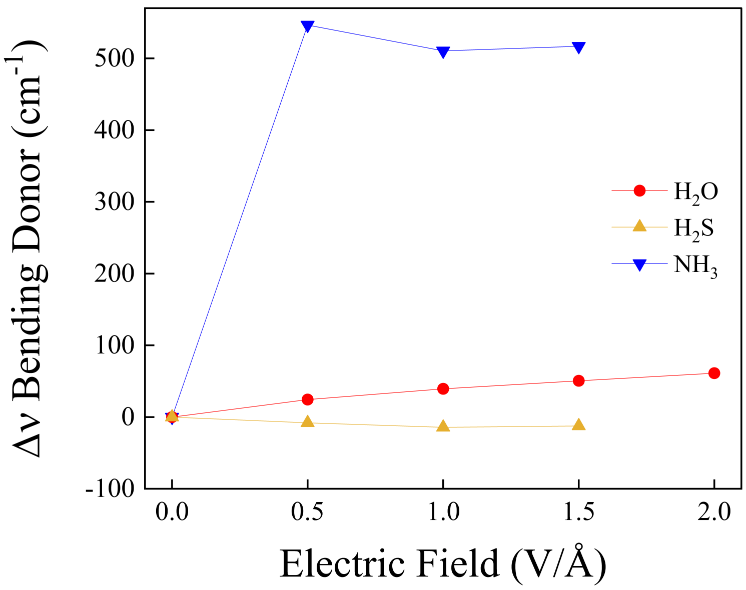

Figure S4: Infrared vibrational Stark effect of the bending mode of the molecular species donating the H-bond in the investigated dimers (see legend) evaluated at the CCSD/aug-cc-pVTZ theory level.

V/Å, respectively. Although the field-induced blue-shift of the bending mode in H-bonded systems is typically associated with the strengthening of the H-bond itself,[4,5] in such a case is simply due to the field-induced locking effect on the NH_3 molecular dipoles in $(NH_3)_2$: even relatively weak fields are capable of freezing molecular vibrations in ammonia, a phenomenon underlying the observed electrofreezing in bulk liquid ammonia samples.[6]

SAPT vs. CCSD(T)/CBS interaction energies

The symmetry-adapted perturbation theory SAPT2+(3)δMP2/aug-cc-pVTZ total interaction energies were compared to the reference CCSD(T)/complete basis set (CBS) interaction energies where aug-cc-pVDZ, aug-cc-pVTZ, and aug-cc-pVQZ basis sets were used for extrapolation to the CBS limit.[7,8] For the CCSD(T) calculations on monomers, EF was applied along the same direction as for the dimers calculations. Results show that the difference between the SAPT and CCSD(T) data is within 0.5 kcal/mol for all the investigated points

(Fig. S5), which justifies the use of the chosen SAPT2+(3)δMP2/aug-cc-pVTZ level of theory for calculations with applied EF.

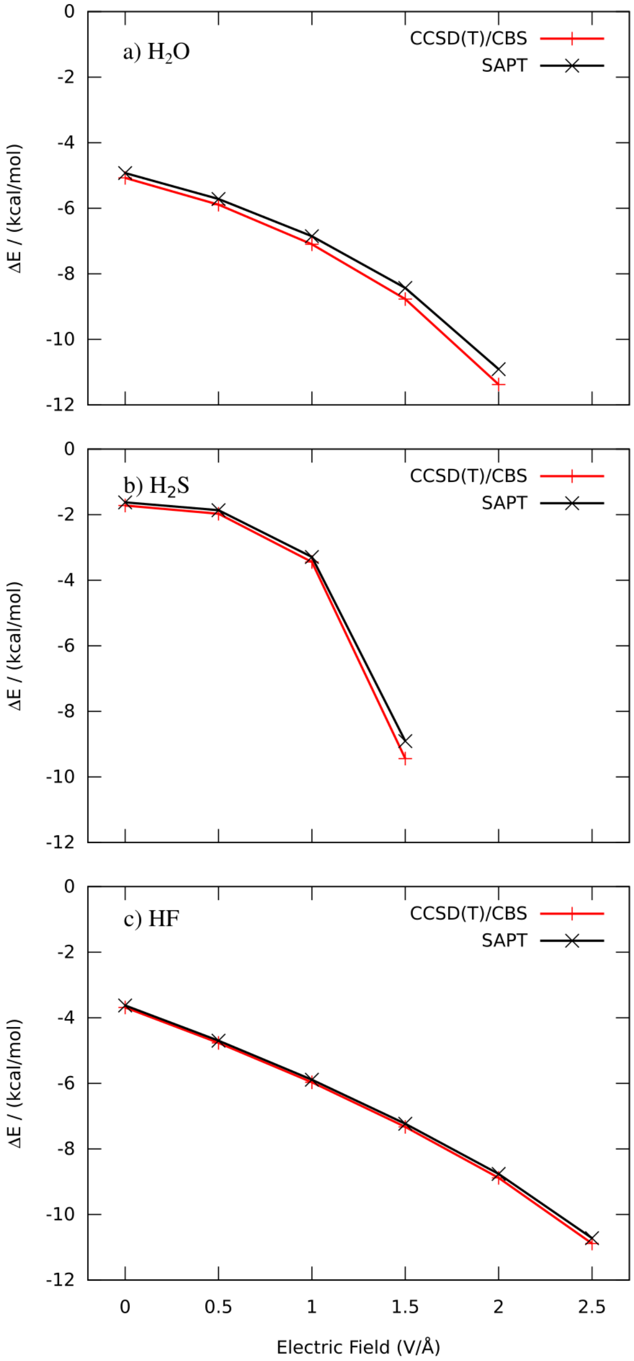

Figure S5: Comparison of the SAPT [SAPT2+(3)δMP2/aug-cc-pVTZ] total interaction energies with the reference CCSD(T)/CBS (cc-pVDZ, cc-pVTZ and cc-pVQZ basis sets were used for extrapolation to complete basis set) interaction energies at 0.0–2.5 V/Å EF intensity.

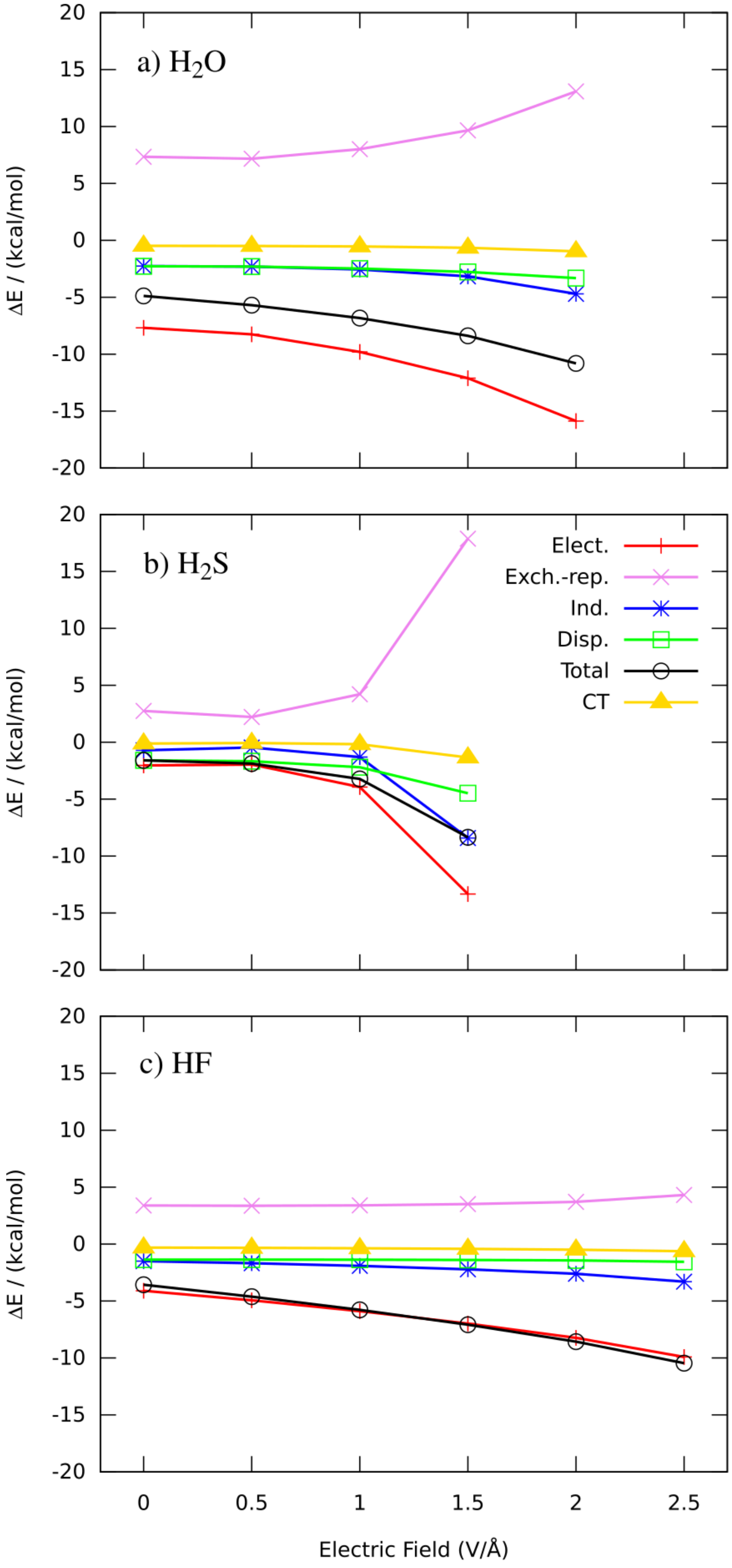

Figure S6: Full SAPT2+(3)-ct/aug-cc-pVQZ profiles including electrostatics (Elect.), exchange-repulsion (Exch.-rep.), induction (Ind.), London dispersion (Disp.), total interaction energy (Total) and energy change related to the charge-transfer (CT) effect. The CT contribution is extracted from the induction term.[9]

HOMO-LUMO Band Gaps

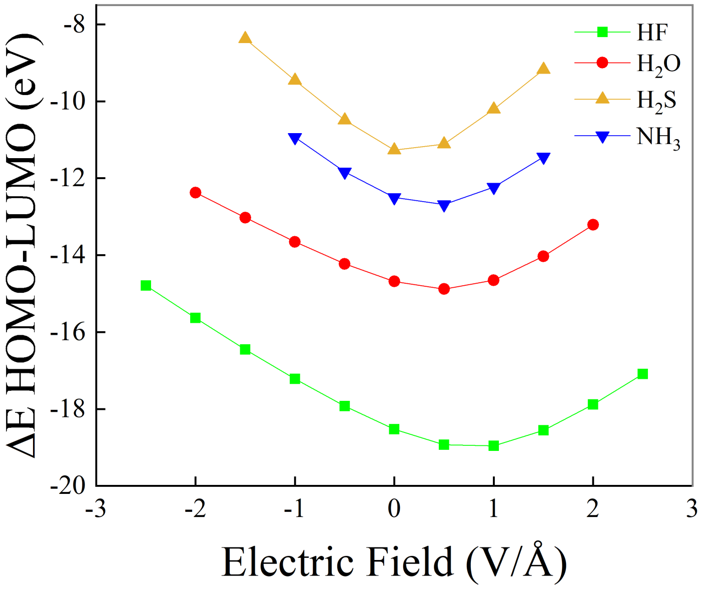

Figure S7: HOMO HOMO-LUMO energy gap of hydrogen fluoride (green squares), water (red dots), hydrogen sulfide (yellow up-triangles), and ammonia (blue down-triangles) for various field intensities and evaluated at the CCSD/aug-cc-pVTZ theory level.

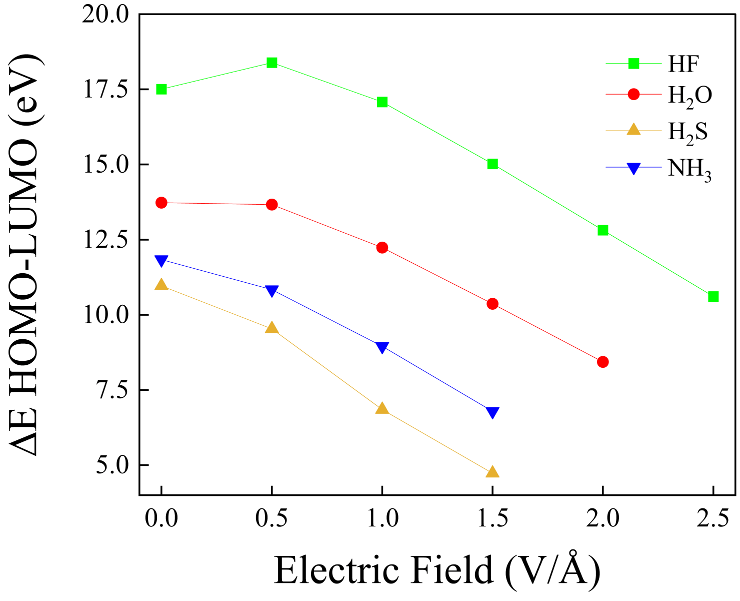

Figure S8: HOMO-LUMO energy gap of the hydrogen fluoride (green squares), water (red dots), and hydrogen sulfide (yellow up-triangles) dimers for various field intensities and evaluated at the CCSD/aug-cc-pVTZ theory level.

Modeling the Stark effect

To obtain the quantities that appear in the right-hand side of Eq (2) (main text) we carried out a series of single-point CCSD/aug-cc-pVTZ calculations on geometries displaced along the selected symmetric stretching and bending normal modes. By using the keyword polar, in addition to the CCSD/cc-pVTZ energy we also obtained the electric dipole and polarizabilty. The numerical results of such calculations are reported in a series of plots in Figs. S9 and S10. To recover the numerical data reported in Table II of the main text, we carried out a polynomial fit expansion of the data, from which the different derivatives could be quickly determined:

$$
\begin{aligned}
E(q_k) &\approx E_0 + \frac{\partial E}{\partial q_k} q_k + \frac{1}{2}\omega_k^2 q_k^2 + \frac{1}{3!} f_{kkk}\, q_k^3 + ... \\
\mu_x(q_k) &\approx \mu_x + \frac{\partial \mu_x}{\partial q_k} q_k + \frac{1}{2}\frac{\partial^2 \mu_x}{\partial q_k^2} q_k^2 + ... \\
\alpha_{xx}(q_k) &\approx \alpha_{xx} + \frac{\partial \alpha_{xx}}{\partial q_k} q_k + \frac{1}{2}\frac{\partial^2 \alpha_{xx}}{\partial q_k^2} q_k^2 + ...
\end{aligned}
\tag{1}
$$

To ensure proper convergence in the lower degrees coefficients, the polynomial numerical fits were carried up to the 5th degree, on a number of 20 data points. The normal modes were computed on very tight geometry optimized structures and the convergence of the SCF and CCSD energy was set at 10^{-13} atomic units (10^{-12} in the case of H_2S). The CCSD normal modes were computed with the numerical Hessian determined with four displacements (instead of two as the default Gaussian's behavior).

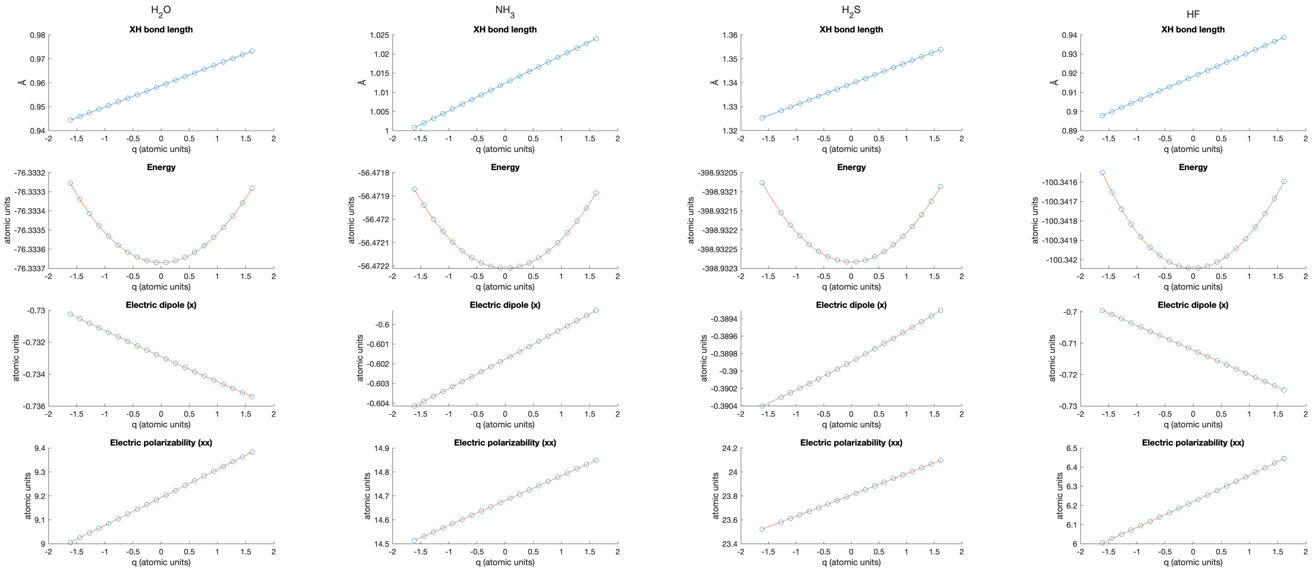

Figure S9: Polynomial fits of the energy, electric dipole and polarizability used to derive the numerical values reported in Table II of the main text. These data are for the stretching modes.

.

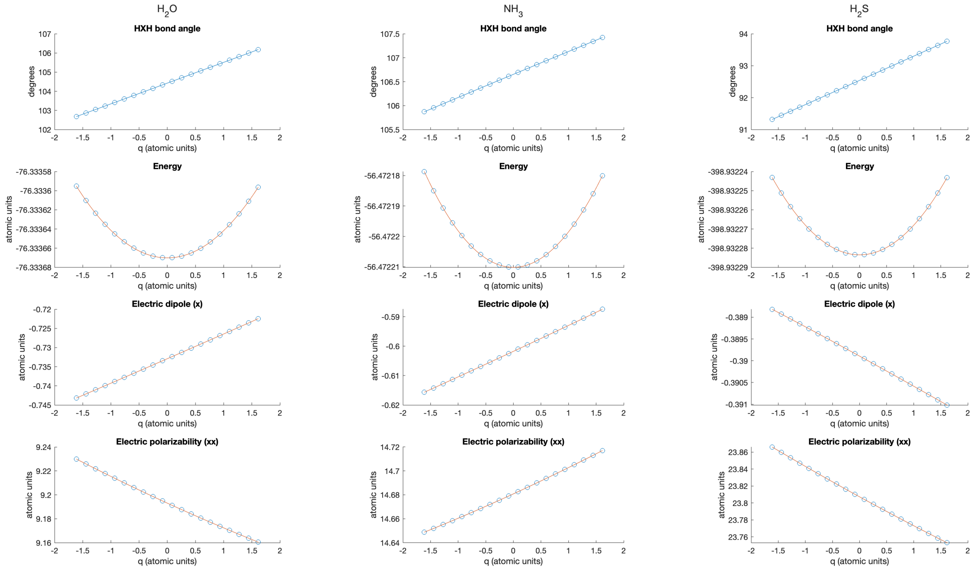

Figure S10: Polynomial fits of the energy, electric dipole and polarizability used to derive the numerical values reported in Table II of the main text. These data are for the bending modes.

.

(a)

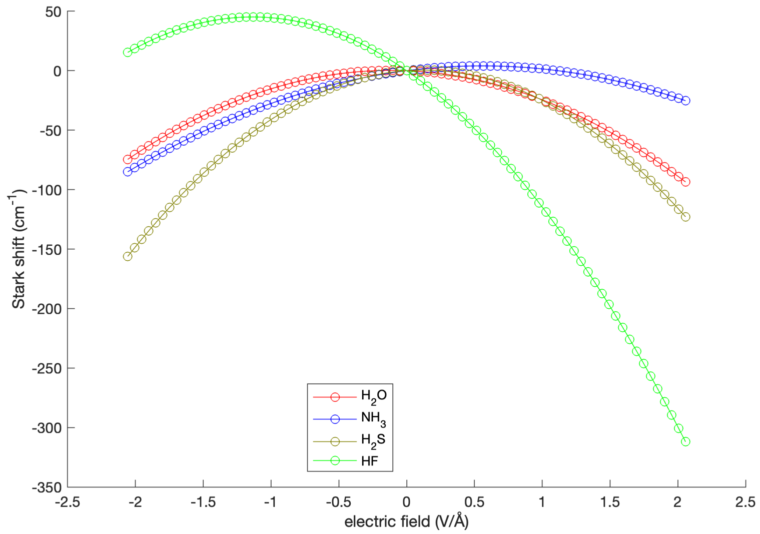

(b)

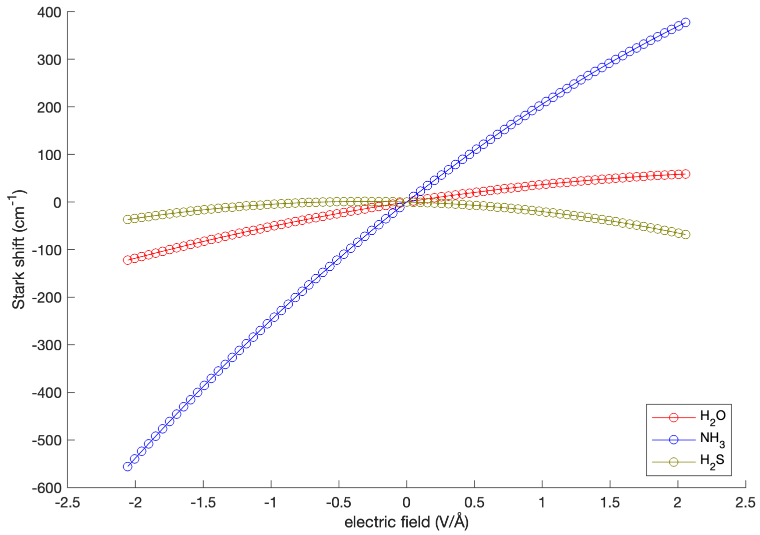

Figure S11: Plots of Bishop's model of the vibrational Stark effect (Eq. 2 of the main text) based on the numerical values reported in Table II of the main text – (a) stretching modes; (b) bending modes

.

References

(1) Frisch, M. J.; Trucks, G. W.; Schlegel, H. B.; Scuseria, G. E.; Robb, M. A.; Cheeseman, J. R.; Scalmani, G.; Barone, V.; Petersson, G. A.; Nakatsuji, H.; et al. Gaussian16 Revision C.01. 2016; Gaussian Inc. Wallingford CT.

(2) Neese, F. The ORCA program system. *WIRES Comput. Molec. Sci.* **2012**, *2*, 73–78.

(3) Jing, A.; Szalewicz, K.; van der Avoird, A. Ammonia dimer: extremely fluxional but still hydrogen bonded. *Nature Communications* **2022**, *13*, 1470.

(4) Cassone, G.; Sponer, J.; Trusso, S.; Saija, F. Ab initio spectroscopy of water under electric fields. *Phys. Chem. Chem. Phys.* **2019**, *21*, 21205–21212.

(5) Cassone, G.; Martelli, F. Electrofreezing of liquid water at ambient conditions. *Nature Communications* **2024**, *15*, 1856.

(6) Cassone, G.; Sponer, J.; Sponer, J. E.; Saija, F. Electrofreezing of Liquid Ammonia. *The Journal of Physical Chemistry Letters* **2022**, *13*, 9889–9894, PMID: 36255376.

(7) Helgaker, T.; Klopper, W.; Koch, H.; Noga, J. Basis-set convergence of correlated calculations on water. *The Journal of Chemical Physics* **1997**, *106*, 9639–9646.

(8) Halkier, A.; Helgaker, T.; Jørgensen, P.; Klopper, W.; Olsen, J. Basis-set convergence of the energy in molecular Hartree–Fock calculations. *Chemical Physics Letters* **1999**, *302*, 437–446.

(9) Stone, A. J.; Misquitta, A. J. Charge-transfer in Symmetry-Adapted Perturbation Theory. *Chemical Physics Letters* **2009**, *473*, 201–205.
